# Genealogical asymmetry under the isolation with migration model and a two-taxon test for gene flow

**DOI:** 10.1093/genetics/iyae157

**Published:** 2024-09-30

**Authors:** Alexander Mackintosh, Derek Setter

**Affiliations:** Department of Ecology and Genetics, Uppsala University, Norbyvägen 18D, Uppsala 75236, Sweden; Institute of Ecology and Evolution, Ashworth Laboratories, University of Edinburgh, Charlotte Auerbach Road, Edinburgh EH9 3FL, UK; Institute of Ecology and Evolution, Ashworth Laboratories, University of Edinburgh, Charlotte Auerbach Road, Edinburgh EH9 3FL, UK

**Keywords:** gene flow, demographic inference, coalescent

## Abstract

Methods for detecting gene flow between populations often rely on asymmetry in the average length of particular genealogical branches, with the ABBA-BABA test being a well-known example. Currently, asymmetry-based methods cannot be applied to a pair of populations and such analyses are instead performed using model-based methods. Here we investigate genealogical asymmetry under a two-population Isolation with Migration model. We focus on genealogies where the first coalescence event is between lineages sampled from different populations, as the external branches of these genealogies have equal expected length as long as there is no post-divergence gene flow. We show that unidirectional gene flow breaks this symmetry and results in the recipient population having longer external branches. We derive expectations for the probability of this genealogical asymmetry and propose a simple statistic ($A_{m}$) to detect it from genome sequence data. $A_{m}$ provides a two-taxon test for gene flow that only requires a single unphased diploid genome from each population, with no outgroup information. We use analytic expectations and simulations to explore how recombination, unequal effective population sizes, bidirectional gene flow and background selection influence $A_{m}$ and find that the statistic provides unambiguous evidence for gene flow under a continent-island history. We estimate $A_{m}$ for genome sequence data from *Heliconius* butterflies and *Odocoileus* deer, generating results consistent with previous model-based analyses. Our work highlights a signal of gene flow overlooked to date and provides a method that complements existing approaches for investigating the demographic history of recently diverged populations.

## Introduction

Hybridization between members of closely related species can sometimes be observed in nature. Depending on the reduction in fitness suffered by early generation hybrids, this process can lead to gene flow between species. Early investigations of the sequence variation within whole genome sequence (WGS) datasets showed that historic gene flow is common, even between species separated by millions of generations of divergence (Kulathinal *et al*. 2009; Martin *et al*. 2013). Over the last decade there has been a considerable increase in the volume of WGS data being generated from natural populations, along with a similar increase in methods for detecting and characterizing gene flow (Hibbins and Hahn 2022). As a result, it is now possible to obtain detailed information about past gene flow and ongoing hybridization within natural systems (see Jensen *et al*. 2023; Satokangas *et al*. 2023; Marcionetti *et al*. 2024 for some recent examples). At the same time, understanding how gene flow shapes patterns of sequence variation, and how this can in turn be used to infer the evolutionary history of populations, is still an active area of research (Cousins *et al*. 2024; Galtier 2024).

### Asymmetry-based methods for detecting gene flow

A natural way to think about how past gene flow can be inferred from WGS data is to consider the genealogical history of individuals sampled from multiple populations. Such a history can be described by the multispecies coalescent (Kingman 1982; Tajima 1983; Takahata *et al*. 1995; Rannala and Yang 2003; Jiao *et al*. 2021). Even under a history of strict bifurcation, the stochasticity of the coalescent process results in individual genealogies that do not match the species history (Tajima 1983; Takahata 1989). Such genealogies are generated when multiple lineages with different sampling locations reach the same ancestral population, with the random order of coalescence events generating alternative topologies with equal probability. Post-divergence gene flow, however, leads to certain topologies being more common than others, and, consequently, to an asymmetry in the average length of particular genealogical branches. The ABBA-BABA test is a well-known approach that uses such genealogical asymmetry to infer gene flow. This four-taxon test was first used to detect historic gene flow between Neanderthals and the ancestors of non-African humans (Green *et al*. 2010) and has since been applied to hundreds of datasets. The test detects past gene flow between nonsister populations, but gives little to no information about the rate, direction, and timing of that gene flow. This has motivated the development of other asymmetry-based methods, that either have improved precision (Martin *et al*. 2015; Lopez Fang *et al*. 2024) or provide more detailed information than the original ABBA-BABA test (Pease and Hahn 2015; Hamlin *et al*. 2020; Martin and Amos 2021). Two major strengths of asymmetry-based approaches are their flexibility and simplicity. For example, the history of gene flow across a sample of tens or even hundreds of populations can be investigated by applying the ABBA-BABA test to all relevant quartets and partitioning the signal across branches of the species tree (Eaton and Ree 2013; Jensen *et al*. 2023). It is also possible to apply asymmetry-based methods across genomes to identify regions with a history of introgression (Malinsky *et al*. 2015; Martin *et al*. 2015). All the while, it is clear what information in the data is used to detect gene flow — asymmetry in polymorphism patterns that are expected to be symmetrical under a coalescent process without gene flow.

Despite the strengths mentioned above, the ABBA-BABA test and other asymmetry-based methods are fundamentally limited by the fact that they only consider a small fraction of the information contained within a sample of genomes (Lohse and Frantz 2014; Martin and Amos 2021). Consequently, these methods cannot distinguish between gene flow and certain scenarios of ancestral population structure (Slatkin and Pollack 2008; Durand *et al*. 2011) or differences in mutation rate between populations (Xiong *et al*. 2022; Frankel and Ané 2023 but see Koppetsch *et al*. 2024).

### Model-based methods for inferring gene flow

An alternative class of methods are those that explicitly model gene flow within a demographic history (Beerli and Felsenstein 1999; Hey and Nielsen 2004). Given a sample of genome sequences from two or more populations, such methods can be used to calculate statistical support for models with and without gene flow. These methods typically also generate estimates of effective migration rate ($m_{e}$) or admixture proportions, along with the other demographic parameters in the model. One attraction of model-based inference is that researchers can obtain a ‘best fitting’ evolutionary history that is (ostensibly) straightforward to interpret, rather than only testing for the presence or absence of gene flow. Additionally, these methods will tend to leverage more of the information contained in genome sequence data than asymmetry-based methods that focus on just a small subset of polymorphism patterns. For these reasons, model-based inference of divergence with gene flow has become common within population genomics, with a wide selection of methods to choose from (e.g. Gutenkunst *et al*. 2009; Rogers 2019; Flouri *et al*. 2020; Excoffier *et al*. 2021).

Given that model-based methods use rich summaries of sequence variation to infer detailed population histories, it is not immediately clear why any researcher would favor an asymmetry-based summary statistic for detecting gene flow, especially if the number of populations is small. However, model-based methods do have several drawbacks. For instance, it is not always straightforward to understand what information in the data provides evidence for gene flow, and more importantly, whether that evidence could instead be the result of bioinformatic artefacts (Shafer *et al*. 2017). The results of model-based inference are also contingent on the finite set of necessarily simple models for which likelihoods can be calculated. If the true demographic history contains processes that cannot be well approximated by simple models, then all models may fit the data poorly (Becquet and Przeworski 2009; Momigliano *et al*. 2021 but see Huang *et al*. 2022).

Recently, Smith and Hahn (2024) used forward simulations to show that commonly used model-based inference methods can erroneously infer gene flow between fully isolated sister populations when the true history includes natural selection. Although the inferred rates of gene flow were very small, their results highlight the problem of fitting simple demographic models that assume selective neutrality when the evolution of real populations is complex and involves multiple selective forces. Smith and Hahn (2024) suggest that asymmetry-based methods are less likely to lead to false inference of gene flow under pervasive natural selection. They point out, however, that asymmetry-based methods cannot currently be applied to datasets containing samples from only two populations.

### Overview

In this work, we investigate how asymmetry in genealogical branch lengths can be used to detect past gene flow between two recently diverged populations, without the need for an outgroup. We use the Isolation with Migration (IM) model (see Methods) as a framework to investigate the properties of genealogies using a small sample of genomes. Our aim, however, is not to use asymmetry in branch lengths to infer parameters of the IM model. Instead, we highlight genealogical asymmetry as a signal of gene flow that can be identified through simple summaries of WGS data.

First, we derive expectations for the length of external branches on genealogies in which the first coalescence event occurs between lineages sampled from different populations, i.e. gene trees that are incongruent with the species tree. We show that the population-specific external branch lengths of such genealogies are equal in the absence of post-divergence gene flow, but that unidirectional gene flow breaks this symmetry. In most practical cases we only have knowledge about mutations, rather than the genealogies themselves. Therefore, as a second step, we derive expected branch lengths conditional on observing a particular type of mutation. We use this to define a simple summary statistic, $A_{m}$, that can be estimated from mutation counts and used as a test for gene flow. We perform simulations to confirm the accuracy of our analytic results as well as to explore how $A_{m}$ is affected by recombination, unequal population sizes, bidirectional gene flow and background selection. Finally, we demonstrate the power of our asymmetry-based two-taxon test for gene flow using two real-world examples with contrasting demographic histories, and we discuss the strengths and weaknesses of this approach with comparison to other methods.

## Methods

### Model

**Fig. 1.**
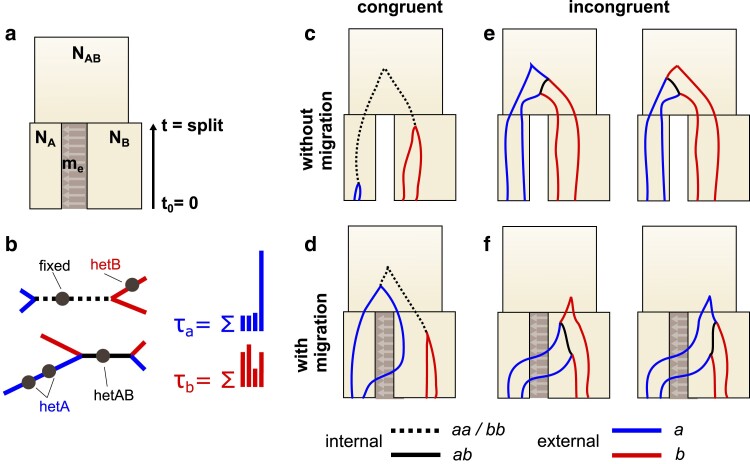
a) The five-parameter IM model includes the sizes of the sampled populations $N_{A}$ and $N_{B}$, a population split time *t*, the ancesteral population size $N_{AB}$, and the effective migration rate $m_{e}$. Here, gray arrows indicate the direction of migration *forward* in time, while $m_{e}$ is the coalescent migration rate *backward* in time. b) Unrooted genealogies may be congruent, having an internal *aa*/*bb* type branch (dashed black), or incongruent, having an internal *ab* type branch (solid black). External branches *a* (in blue) and *b* (in red) distinguish samples from the *A* and *B* populations respectively. $\tau_{A}$ and $\tau_{B}$ are the total branch length of *a* and *b* type branches, respectively. Mutations on the internal branches of genealogies lead to shared heterozygous sites (*hetAB* polymorphisms) when the topology is incongruent, and fixed differences (*fixed* polymorphisms) when the topology is congruent. Mutations on the external branches result in polymorphisms in just one population (*hetA* and *hetB*), no matter the topology. c and d) Congruent genealogies are generated whenever the first coalescence event is between lineages sampled from the same population. e and f) Incongruent genealogies, where the first coalescence event is between lineages sampled from different population, can be generated through failed coalescence (e) or migration (f).

We focus on an IM model with five parameters (Fig. 1a). The parameters $N_{A}$, $N_{B}$, and $N_{AB}$ correspond to the effective sizes of populations *A*, *B*, and the ancestral population, respectively. The parameter *t* is the number of generations between the present and the onset of divergence, and $m_{e}$ is the effective rate of migration (pastward) between populations *A* and *B*. Initially, we only consider unidirectional migration, with $m_{e}$ representing the rate at which lineages migrate from population *A* to population *B* backwards in time, with no migration in the opposite direction.

We consider a sample of two lineages (equivalently, a diploid individual) from each population (Fig. 1). Genealogies from this sampling scheme contain four leaves, and if leaves are labeled by the population from which they are sampled (either *a* or *b*), there are two possible unrooted topologies: those in which the first coalescence event is a+a or b+b (congruent with the species tree), and those in which the first coalescence event is a+b (incongruent) (Fig. 1b). We identify branches on these genealogies as either external or internal, with external branches defined as those leading directly to a leaf on the unrooted genealogy. Labeling branches by their descendants results in external branches with the labels *a* and *b*, and internal branches with the labels *aa* (equivalent to *bb* on an unrooted tree) and *ab* (Fig. 1b). The total length of branch types ($\tau_{a}$, $\tau_{b}$, $\tau_{aa}$, $\tau_{ab}$) are (nonindependent) random variables with a complicated joint distribution determined by the coalescent process (note that some previous work has used the notation *t* rather than *τ*). For simplicity, we focus only on the marginal distributions of the total external branch lengths ($\tau_{a}$ and $\tau_{b}$), particularly their expected values (${\bar{\tau }}_{a}$ and ${\bar{\tau }}_{b}$).

### Coalescent simulations

In the Results section, we define two statistics ($A_{i}$ and $A_{m}$) that are summaries of the total external branch lengths $\tau_{a}$ and $\tau_{b}$. We are interested in how these summary statistics behave under different scenarios of population divergence, in particular with and without gene flow. In addition to analytic results, we used msprime 1.3.0 to simulate population histories under the IM model described in the previous section (Baumdicker *et al*. 2021). The general simulation procedure includes populations of size $N_{e}=100,000$, a split time of t=200,000, $m_{e}\in \{0,5.0\times 10^{-6},1.5\times 10^{-5}\}$ (i.e. $2Nm_{e}\in \{0,1,3$}), and a mutation rate of $\mu =1\times 10^{-8}$ per-site per-generation. When simulations differ from these parameters, or include additional processes such as recombination, we state this in the relevant Results section. We recorded external branch lengths of simulated genealogies (i.e. $\tau_{a}$ and $\tau_{b}$) in short blocks of sequence using tskit 0.5.6, and used these values to estimate the summary statistics $A_{i}$ and $A_{m}$. We used a block-jackknife resampling procedure to estimate 95% confidence intervals of $A_{m}$ (Quenouille 1949; Malinsky *et al*. 2021). To limit the computation time of the block-jackknife we grouped sequence blocks into jackknife-blocks (minimum of 25), ensuring that nonindependent sequence blocks from the same simulation replicate were grouped together. Code for performing the coalescent simulations is provided as a python notebook (see Data availability).

### Forwards simulations and comparison with other methods

We simulated recombining genomes with background selection (BGS) using SLiM 4.2.2 (Haller *et al*. 2019; Haller and Messer 2023) to investigate the behavior of the summary statistic $A_{m}$ (and other methods for detecting gene flow) under natural selection. Genomes consisted of 20×5 Mb chromosomes. Each chromosome had the same U-shaped recombination map (Mb sized windows with rates: $1.6\times 10^{-8}$, $0.8\times 10^{-8}$, $0.2\times 10^{-8}$, $0.8\times 10^{-8}$, $1.6\times 10^{-8}$) and a mean rate of $1\times 10^{-8}$ per-base per-generation. Genomes contained 5,000 genic regions of length 2 kb, each centred within a 20 kb interval. Deleterious mutations occurred within genic regions at a rate of $1\times 10^{-8}$ per-site per-generation, and selection coefficients were sampled from an exponential distribution with mean −0.01. To allow for the possibility of associative overdominance, the dominance coefficient of deleterious mutations was set to h=0.25, as in Smith and Hahn (2024). Given a population size of N=10,000, this distribution of fitness effects has a mean 2Nsh of −50.

We simulated 100 replicates of three different scenarios. The first scenario (SI) involves a single population (P1) of N=10,000 diploids evolving for 8N (burn-in) generations, followed by populations (again N=10,000) splitting off without gene flow at times 8N, 14N and 15N generations, with the sampling time at 16N generations. This generates a population branching topology of (((P1, P2), P3), O), with splits times of 10,000, 20,000 and 80,000 generations ago for populations P1-P2, P1-P3, and P1-O, respectively. The second scenario (SI with unequal *N*) is the same as the first except that the population size of P3 is N=20,000. The third scenario (gene flow) has equal population sizes but undirectional gene flow from P3 to the common ancestor of (P1, P2) and then gene flow only to P2 ($m_{e}=2.5\times 10^{-5}$).

Tree sequences were recapitated with pyslim 1.0.4 and msprime, and then simplified to include five diploids randomly sampled from each population. Neutral mutations were added to the tree sequence with a rate of $1\times 10^{-8}$ per-site per-generation and nongenic regions were sampled for downstream analysis. Variants were written to a VCF file and $A_{m}$ was calculated using the script estimate_Am.py (see Data availability), focusing only on samples from P2 and P3. We used a block size of 201 bases and chromosomes were used as jackknife blocks. Similarly, the D-statistic of the ABBA-BABA test was calculated with Dsuite (Malinsky *et al*. 2021), using samples from all four populations and 20 jackknife blocks. We conditioned the D-statistic calculation on the simulated population branching order, as otherwise the order ((P2, P3), P1) was often assumed. The joint site frequency spectrum (jSFS) of P2 and P3 was directly summarized from mutations in the tree sequence using tskit, while assuming knowledge of the ancestral allelic state at each SNP. We fit an IM model with bidirectional gene flow to the jSFS using moments (Jouganous *et al*. 2017). We fit the full model as well one where $m_{P2}=m_{P3}=0$, and reported both $m_{e}$ values as zero whenever the latter model fit better.

### Analysis of real data

We reanalyzed WGS data from *Heliconius* butterflies and *Odocoileus* deer. More specifically, we used the filtered VCF file from Laetsch *et al*. (2023) (shared via personal communication), and converted the genotypes from Kessler *et al*. (2023) (shared via personal communication) to a VCF file with the script genoToVCF.py (Martin and Amos 2021). We estimated $A_{m}$ across a range of block sizes from these VCF files using the script estimate_Am.py (see Data availability). For the *Odocoileus* deer, we analyzed the individuals Ov_ON6 and Oh_WAI, as these are individuals with high sequencing coverage that were also used in the MSMC-IM analysis in Kessler *et al*. (2023). For the *Heliconius* butterflies, we selected individuals ros.CJ2071.m and chi.CJ565.m as they were both male and had high sequence coverage. Jackknife blocks corresponded to $10^{6}$ consecutive SNPs for the *Odocoileus* deer (with blocks sometimes spanning multiple sequence scaffolds), and chromosomes for the *Heliconius* butterflies.

### Parametric bootstraps

We performed a parametric bootstrap analysis to gain further information about the results obtained in *Heliconious* butterflies and *Odocoileus* deer. Each replicate included simulation of a 66 kb sequence under a specific demography. The sequence was then split up into blocks ranging from $2^{0}$ (1) to $2^{15}$ (65,536) bases in length. We used the same strategy as above to calculate point estimates and 95% confidence intervals of $A_{m}$ for each block size.

For the butterflies we performed 1,000 replicate simulations under the IM demography of Laetsch *et al*. (2023). Here, *H. melpomene* ($N_{mel}=549,000$) and *H. cydno* ($N_{cyd}=1,415,000$) split from a common ancestor ($N_{anc}=927,900$) 4,216,000 generations ago. Backward in time, migration occurs from *H. mel* to *H. cyd* at rate $m_{e}=7.4\times 10^{-7}$. Recombination occurs at rate $r=1.89\times 10^{-8}$ (Davey *et al*. 2017); mutation, at rate $\mu =2.9\times 10^{-9}$ (Keightley *et al*. 2015) (see python notebook 2).

For the deer, we ran 1,000 replicate simulations of the three-population demography from Fig. 4a in Kessler and Shafer (2024), as well as 1,000 replicates of the alternative demography in Fig. 4b. Both models share the recombination rate $r=1.04\times 10^{-8}$ (Johnston *et al*. 2017) and mutation rate $\mu =1.23\times 10^{-8}$ (Chen *et al*. 2019), as well as the property of bidirectional gene flow. The full parameters of these two demographic models are given in Table S5 of Kessler and Shafer (2024). Given their complexity, we do not describe the models here but code for simulating them can be found in python notebooks 3 and 4 (see Data availability).

## Results

### Genealogical incongruence and asymmetry

Under the IM model, the total (marginal) length of external branches *a* and *b* ($\tau_{a}$ and $\tau_{b}$) depend on the split time *t*, migration rate $m_{e}$, and the three population sizes $N_{A}$, $N_{B}$, and $N_{AB}$ (Figure 1a), and in general, we do not expect ${\bar{\tau }}_{a}={\bar{\tau }}_{b}$. Equality of the expected branch lengths only occurs under two scenarios: when there is no divergence (i.e. t=0) or when there is divergence without gene flow between two equal-sized populations (i.e. t>0 and $m_{e}=0$ with $N_{A}=N_{B}$). In other words, for any biologically realistic case of population divergence we expect ${\bar{\tau }}_{a}\neq {\bar{\tau }}_{b}$ (Fig. 1c and d). However, if one focuses exclusively on genealogies with an incongruent topology (Fig. 1e and f), ${\bar{\tau }}_{a}\neq {\bar{\tau }}_{b}$ is an unambiguous indicator of post-divergence gene flow between the two populations. We can understand this as follows.

Incongruence can be generated two ways: either failed coalescence in populations *A* and *B* or through gene flow. Assuming no gene flow ($m_{e}=0$), incongruence occurs solely due to failed coalescence and with probability


(1) $$P(incongruence\;|\;m_{e}=0)=\frac{2}{3}e^{-t/2N_{A}}e^{-t/2N_{B}}.$$

For such genealogies, all four lineages must be present in the ancestral population and the order in which they coalesce is random (Fig. 1e). Conditioned on incongruence, the expected value of the (marginal) total branch length distributions is


(2) $$E[\tau_{a}\;|\;m_{e}=0]=E[\tau_{b}\;|\;m_{e}=0]=2t+\frac{8}{3}N_{AB}$$

The fact that equation (2) applies to both *a* and *b* branches and is independent of $N_{A}$ and $N_{B}$ means that, on-average, genealogies with an incongruent topology are expected to have equal external branch lengths (${\bar{\tau }}_{a}={\bar{\tau }}_{b}$) when $m_{e}=0$ even if populations *A* and *B* have drastically different effective population sizes (Fig. 1e). Although this result is not particularly surprising, it is important in that it provides an expectation for symmetry in external branch lengths (conditional on incongruence) under a two-population history without gene flow.

Given the result above, we next investigate whether post-divergence gene flow breaks the symmetry in external branch lengths of incongruent genealogies. For simplicity, we now assume that all three populations share the same $N_{e}$ and use the parameters $M=2N_{e}m_{e}$ and $T=t/2N_{e}$ for ease of notation. Lohse *et al*. (2016) derived marginal distributions of branch lengths $\tau_{a}$, $\tau_{b}$, $\tau_{aa}$, and $\tau_{ab}$ under the IM model, showing that unidirectional gene flow does indeed generate asymmetry between $\tau_{a}$ and $\tau_{b}$ (see their Fig. 5). The results of Lohse *et al*. (2016) apply to a random sample of genealogies, without any conditioning on topology. As a result, asymmetry between $\tau_{a}$ and $\tau_{b}$ is not only generated by gene flow but also by differences in $N_{e}$ between the two populations. Here we are interested in properties of incongruent genealogies where asymmetry between $\tau_{a}$ and $\tau_{b}$ can only be generated by gene flow. We therefore use the same approach as Lohse *et al*. (2016)—the generating function (GF) of genealogical branches (Lohse *et al*. 2011)—but condition on observing an incongruent genealogy.

A key feature is that the GF is a series of terms, each representing a unique sequence of coalescence, migration, and population split events. We refer to these as the *paths* of the IM model, of which there are 95. For example, the path for the genealogy in Fig. 1d is defined by the following events (looking pastward in time): migration from *A* to *B*, coalescence of the *b* lineages, the population split, coalescence of the *a* lineages, and finally coalescence of the *aa* and *bb* lineages. By extracting the 33 terms in the GF that correspond to paths with an incongruent topology (i.e. those that contain *ab* branches), the probability of incongruence can be calculated as:


(3) $$P(incongruence)=\frac{2e^{-2T-2TM}+2M}{3+3M}$$

(see Equation 13 of Lohse *et al*. 2016). Setting M=0 recovers equation (1), while M>0 yields a higher probability of observing an incongruent genealogy.

**Fig. 2.**
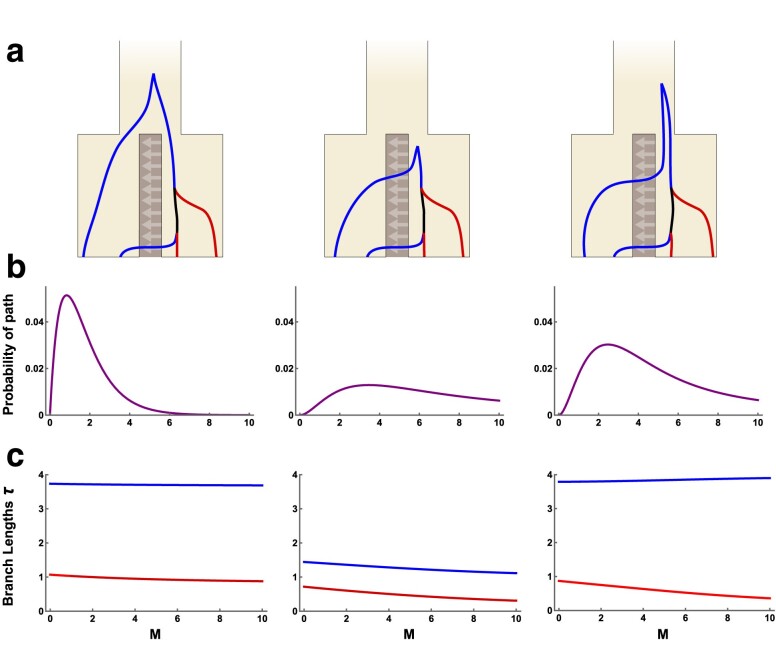
The three asymmetrical incongruent genealogies (a) are generated when the first event is migration, the second event is *a*+*b* coalescence, and the third event is *ab*+*b* coalescence with the remaining *a* lineage still isolated in population *A*. Here, blue and red correspond to the external branches for samples from the *A* and *B* population, respectively (see Fig. 1). Each genealogy has a unique probability of occurring (b) and a different disparity in the expected total length of external branches (c), both of which depend on the split time (here, T=1.0) and the migration rate *M* (*x*-axis). Branch lengths $\tau_{a}$ and $\tau_{b}$ are shown in blue and red, respectively, and in units of $2N_{e}$ generations. In all plots, $N_{A}=N_{B}=N_{AB}$.

We can gain information about how external branch lengths are affected by gene flow by obtaining the expected values of $\tau_{a}$ and $\tau_{b}$ independently for each of the 33 incongruent paths in the GF. Of these paths, 10 have expected branch lengths with ${\bar{\tau }}_{a}={\bar{\tau }}_{b}$. Another 20 have expected branch lengths where ${\bar{\tau }}_{a}\neq {\bar{\tau }}_{b}$, but each is an equiprobable member of a pair, for which the combined branch lengths of *a* and *b* are equal (e.g. Fig. 1e and f). This is because the members of each pair are exchangeable but for the order of the last two coalescence events. The three remaining paths are unpaired and have expected branch lengths ${\bar{\tau }}_{a}>{\bar{\tau }}_{b}$. It is these three paths, shown in Fig. 2a, that break the symmetry in external branch lengths on incongruent genealogies when M>0. We hereafter refer to these genealogies as asymmetrical. In the same figure, we show the probability of each such genealogical history (Fig. 2b) as well as the expected values of $\tau_{a}$ and $\tau_{b}$ (Fig. 2c) in relation to *M* (here, assuming T=1). These results show that the probability of asymmetrical genealogies peaks at intermediate values of *M* (Fig. 2b), but that the difference in ${\bar{\tau }}_{a}$ and ${\bar{\tau }}_{b}$ remains substantial across *M* values (Fig. 2c). Note as well that these asymmetrical incongruent genealogies are only observed when there is gene flow, i.e. they have zero probability when M=0 (Fig. 2b).

**Fig. 3.**
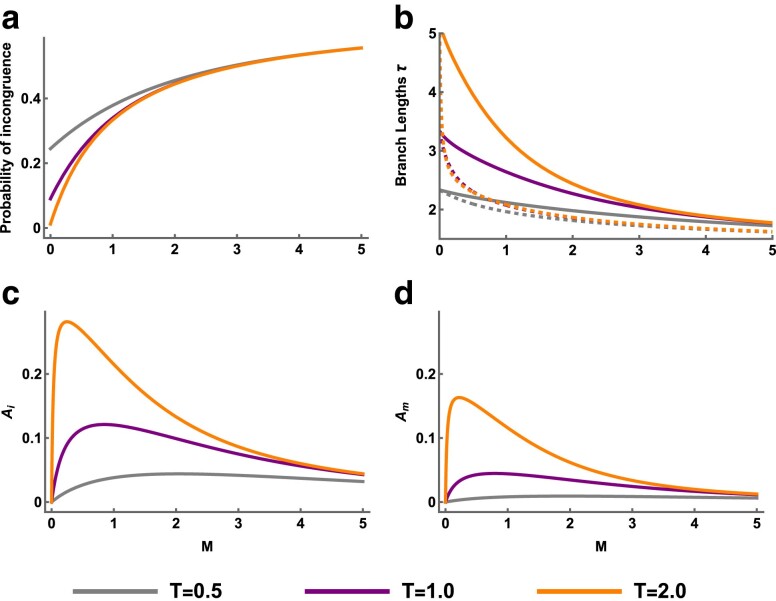
The effect of migration rate *M* and divergence time *T* on a) the probability of incongruence, b) the expected total length of external branches on incongruent genealogies, c) the scaled difference in external branch lengths on incongruent genealogies ($A_{i}$) and d) the scaled difference in external branch lengths conditional on observing a *hetAB* mutation in a 200 bp block of sequence ($A_{m}$), given a mutation rate of $1\times 10^{-8}$ per-site per-generation and an $N_{e}$ of 100,000 in all populations. Gray, purple, and orange lines correspond to divergence times T={0.5,1.0,and2.0}, respectively. In panel (b), the solid lines correspond to ${\bar{\tau }}_{a}$, the dashed lines to ${\bar{\tau }}_{b}$, and branch lengths are measured in units of $2N_{e}$ generations.

The above results suggest that, across incongruent genealogies, the asymmetry of *a* and *b* branch lengths can be used to distinguish between divergence with and without gene flow. Additionally, the relative values of $\tau_{a}$ and $\tau_{b}$ also provide information about the direction of gene flow, as the recipient population (forwards in time) is expected to have longer external branches. We next calculate expectations for $\tau_{a}$ and $\tau_{b}$ conditional on sampling a random incongruent genealogy, rather than a specific path. Figure 3 shows the probability of incongruence as well as the ${\bar{\tau }}_{a}$ and ${\bar{\tau }}_{b}$ of incongruent genealogies, dependent on *M* and *T*. Additionally, we summarize the difference in external branch lengths on incongruent genealogies using the scaled ratio


(4) $$A_{i}=\frac{{\bar{\tau }}_{a}-{\bar{\tau }}_{b}}{{\bar{\tau }}_{a}+{\bar{\tau }}_{b}},$$

where ${\bar{\tau }}_{a}={\bar{\tau }}_{b}$ leads to $A_{i}=0$, ${\bar{\tau }}_{a}>{\bar{\tau }}_{b}$ to $A_{i}>0$ and ${\bar{\tau }}_{b}>{\bar{\tau }}_{a}$ to $A_{i}<0$. Intermediate values of *M* generate the greatest inequality in ${\bar{\tau }}_{a}$ and ${\bar{\tau }}_{b}$, and therefore large values of $A_{i}$ (Fig. 3c). This can be explained by the fact that asymmetrical genealogies are only generated when just one lineage migrates before the first coalescence event (Fig. 2). As the divergence time *T* increases, so does $A_{i}$ (Fig. 3c). With no divergence (T=0) all four lineages are effectively sampled from the ancestral population and so $A_{i}=0$. By contrast, a migration only model (T=∞) eliminates the possibility that the first event is the merging of populations *A* and *B*, thereby maximizing both the frequency of asymmetrical genealogies and $A_{i}$.

**Fig. 4.**
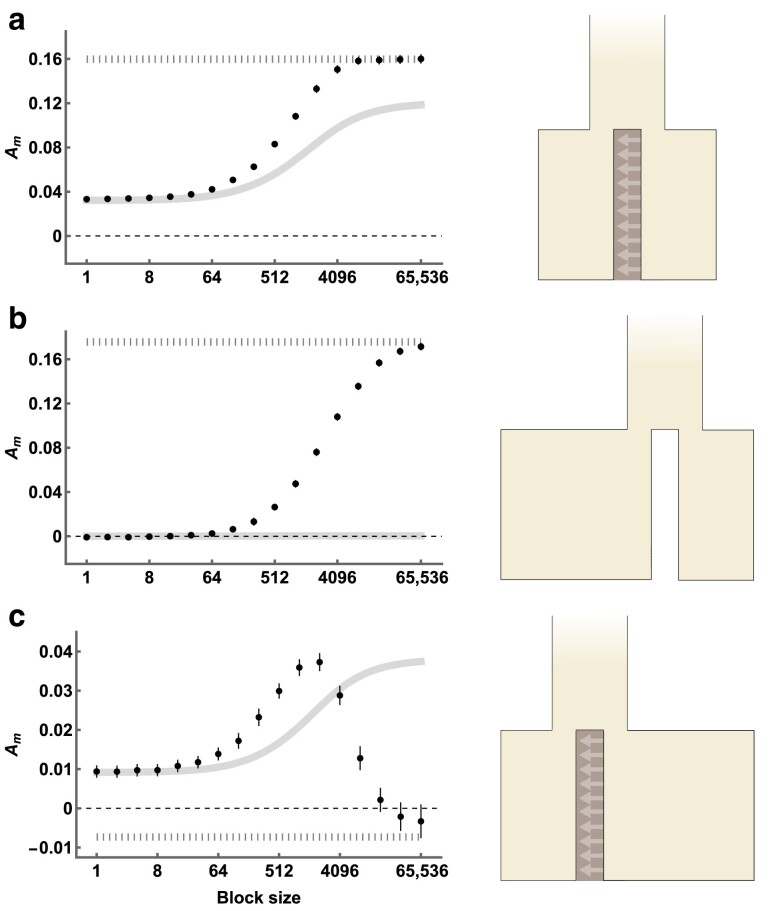
Estimates of $A_{m}$ for various block sizes for simulations with recombination and a demographic model with a) Equal population sizes $N_{A}=N_{B}=N_{AB}=100,000$ and unidirectional migration at rate M=1.0 from population *B* to *A* (forwards in time), b) Unequal population sizes $N_{A}=200,000$ and $N_{B}=N_{AB}=100,000$ with strict isolation M=0, and c) Unequal population sizes $N_{A}=N_{AB}=100,000$ and $N_{B}=200,000$ with unidirectional migration from *B* to *A* forwards in time at rate M=1.0. In all panels, the divergence time T=1.0, the per-base mutation rate $\mu =10^{-8}$, and the recombination rate $r=10^{-8}$ per-base per-generation. The analytic expectation for $A_{m}$ in the absence of recombination is shown in a thick solid line, whereas the analytic expectation for $A_{m}$ under free recombination is shown as a thick dashed line. Error bars correspond to the 95% CIs of each estimate.

For the biologically relevant range of parameters we consider in Fig. 3, we observe a maximum $A_{i}$ of 0.282. In principle, $A_{i}$ can be as high as 1.0, but such high values are only expected when *T* is large and *M* is small. For example, when T=10 and $M=1\times 10^{-4}$, $A_{i}=0.775$, but the probability that any genealogy is incongruent is extremely low ($6.7\times 10^{-5}$). With so few incongruent genealogies from which to estimate $A_{i}$, we do not expect to be able to use $A_{i}$ for inference under this demography. However, we expect $A_{i}$ to be a useful summary statistic for detecting gene flow between recently diverged populations where incongruent genealogies are common (Fig. 3).

### Estimating asymmetry from patterns of mutation

Given that asymmetry in external branch lengths on incongruent genealogies provides evidence for historic gene flow, how can we identify such genealogies in genome sequence data? Put differently, how can we estimate $A_{i}$? One approach would be to reconstruct the ancestral recombination graph (ARG) for a sample of genomes, and then summarize external branches across the ARG to calculate $A_{i}$. While possible (see Discussion), we instead take a methodologically simpler approach that uses only a single diploid sample from each population and requires neither phase information nor polarization.

For this sampling scheme (an unphased and unpolarized diploid from each population), there are four types of polymorphisms corresponding to the distinct branches of the genealogy: *hetA*, *hetB*, *hetAB*, *fixed* (here, following the nomenclature of Laetsch *et al*. 2023). Assuming an infinite sites mutation model, a *hetAB* polymorphism (in which both diploids are heterozygous for the same alleles) can only be generated when the underlying genealogy is incongruent (Fig. 1b). These mutations mark the approximate locations of a subset of the incongruent genealogies throughout the genome, and we can modify our estimator of genealogical asymmetry by restricting ourselves to those genealogies. We define


(5) $$A_{m}=\frac{{\bar{\tau }}_{a}^{\prime }-{\bar{\tau }}_{b}^{\prime }}{{\bar{\tau }}_{a}^{\prime }+{\bar{\tau }}_{b}^{\prime }}\approx \frac{k_{hetA}^{\prime }-k_{hetB}^{\prime }}{k_{hetA}^{\prime }+k_{hetB}^{\prime }},$$

where ${\bar{\tau }}_{a}^{\prime }$ and ${\bar{\tau }}_{b}^{\prime }$ are the expected external branch lengths of genealogies containing at least one mutation on the *ab* branch. This can be approximated by the counts $k^{\prime }$ of *hetA* and *hetB* polymorphisms that lie within *l* bases of a *hetAB* polymorphism, where a block size of 2l+1 is chosen to be small enough to limit the probability that the region spans more than one genealogy.

In the previous section, we defined $A_{i}$ as a ratio between the expected *a* and *b* branch lengths of incongruent genealogies. $A_{m}$ instead estimates the ratio of *a* and *b* branch lengths in the context of genealogies with at least one *hetAB* polymorphism. This is a nonrandom subset of incongruent genealogies because those with longer internal branches are more likely to carry a *hetAB* mutation. We therefore expect values of $A_{i}$ and $A_{m}$ to be highly correlated but to differ in scale (Fig. 3d). To resolve this discordance, we again use the GF approach and derive the expected branch lengths ${\bar{\tau }}_{a}^{\prime }$ and ${\bar{\tau }}_{b}^{\prime }$ (and therefore $A_{m}$), this time conditioning on those genealogies with at least one *hetAB* mutation. $A_{m}$ now depends on the rate of mutation on the *ab* branch which is parameterized by a per-site mutation rate *μ* and locus length 2l+1. The GF conditioned on observing ≥1*hetAB* mutations is obtained by (i) deriving the GF over which the number of *hetAB* mutations is marginalized (that is, accounts for any number of *hetAB* mutations) then (ii) subtracting the GF conditioned on observing exactly $k_{hetAB}=0$ mutations (for details see Supplementary File 1).

We used coalescent simulations to confirm the accuracy of our derivations of $A_{i}$ and $A_{m}$. Specifically, we simulated divergence with gene flow under IM models with parameters $N_{A}=N_{B}=N_{AB}=100,000$, T=1.0, M∈{0,1.0,3.0} and $\mu =10^{-8}$. We simulated 500,000 nonrecombining sequence blocks of size 200 bases and sampled a single diploid from each population. The values of $A_{i}$ and $A_{m}$ estimated from the branch lengths of simulated genealogies closely match to our analytic expectations (Table 1). Reassuringly, the 95% confidence intervals (95% CIs) for estimates of $A_{m}$ do not span zero when M>0, showing that the effect of gene flow on genealogical asymmetry can be distinguished from a null-scenario given sufficient information about branch lengths.

**Table 1.** Estimates (est.) of $A_{i}$ and $A_{m}$ calculated from simulated genealogies are presented for three different demographic histories.

*M*	est. $A_{i}$ (95% CIs)	pre. $A_{i}$	est. $A_{m}$ (95% CIs)	pre. $A_{m}$
0.0	− 0.00046 (−0.00386, 0.00293)	0.00000	− 0.00190 (−0.00682, 0.00302)	0.00000
1.0	0.12109 (0.11865, 0.12352)	0.12016	0.04260 (0.03865, 0.04656)	0.04394
3.0	0.07569 (0.07299, 0.07838)	0.07494	0.02180 (0.01766, 0.02594)	0.02432

Analytic predictions (pre.) are also given for the same demographic histories. Each estimate is derived from 500,000 simulation replicates of a 200 bp sequence without recombination. The three demographic histories share all parameters ($N_{A}=N_{B}=N_{AB}=100,000$, T=1.0, and $\mu =10^{-8}$) except the rate of migration (M∈{0,1.0,3.0}). For simulation derived estimates of $A_{i}$ and $A_{m}$, 95% CIs are given in brackets and were estimated through block jackknife resampling.

### Recombination and unequal effective population sizes

So far, our analytic and simulated results have assumed that a short block of sequence is associated with a single genealogy. In reality, short blocks of sequence centered on a *hetAB* polymorphism will sometimes reflect multiple genealogies due to recombination, and this has the potential to bias our summary statistic ($A_{m}$) away from its expected value. This is because recombination violates the assumption that nearby *hetA* and *hetB* polymorphisms are on the same incongruent genealogy as the focal *hetAB* polymorphism. Estimates of $A_{m}$ in the limit of high recombination would reflect the average asymmetry in external branch lengths across all genealogies rather than just those that are incongruent, and it would no longer be a valid test for gene flow. It is less clear what to expect when calculating $A_{m}$ from short blocks of sequence (i.e. a few hundred bases) in which recombination only happens occasionally.

To understand the effect of recombination on estimates of $A_{m}$, we simulated population divergence with gene flow as above with M=1.0 but with the addition of recombination at a rate of $r=10^{-8}$ per-base per-generation. We simulated sequences of 66 kb (5,000 replicates) and estimated $A_{m}$ from genealogical branch lengths using different block sizes and therefore varying frequencies of recombination. Note that these block sizes include a single base, which is possible for simulated genealogies with known branch lengths but not when analyzing real data. We find that simulated values of $A_{m}$ closely match the analytic expectation (which assumes no recombination) for very small blocks but then diverge to greater values once the block size reaches around 100 bases (Fig. 4a). At large block lengths, e.g. 16 kb, estimates of $A_{m}$ reach the expectation for asymmetry in external branch lengths given a random sampling of genealogies. These results show that too large a block length can lead to the inclusion of congruent genealogies through recombination and therefore biased estimates of $A_{m}$.

Next, we simulated a history of divergence with strict isolation (M=0), but with inequality in $N_{e}$ between the sampled populations ($N_{A}=200,000$ and $N_{B}=100,000$). We performed the same analysis as above and find that $A_{m}=0$ at short block sizes, as expected under a history of strict isolation (Fig. 4b). However, as the block size increases $A_{m}$ becomes significantly greater than zero and eventually reaches the value expected for random sampling (Fig. 4b). In this case, recombination and unequal $N_{e}$ leads to a false-positive signal of gene flow. This is driven by the inclusion of congruent genealogies, via recombination, where external branch lengths are determined by $N_{A}$ and $N_{B}$ (Fig. 1c). Although this risk can be minimized by choosing a very short block length, thus reducing the frequency of recombination within blocks, this also reduces the number of linked polymorphisms and therefore the power to detect asymmetry in branch lengths. Recombination in the presence of unequal $N_{e}$ therefore represents a challenge for detecting gene flow through asymmetry alone.

Finally, we consider a demographic history where $A_{m}$ is expected to be positive in the absence of recombination, due to gene flow (M=1.0), but $A_{m}$ in the limit of high recombination is expected to be negative due to population *B* being larger in effective size ($N_{A}=N_{AB}=100,000$ and $N_{B}=200,000$). At very small and very large block sizes, simulations match these expectations (Fig. 4c). Surprisingly, the transition between these limits is nonmonotonic with an initial increase in $A_{m}$ after increasing blocks to a few hundred bases in length. To understand this, we performed additional simulations of the same demographic history, with and without migration, and measured the contribution of incongruent and congruent genealogies to $A_{m}$ separately. As expected, short blocks of sequence conditioned on a *hetAB* polymorphism almost exclusively contain incongruent genealogies (Supplementary Fig. 1 panels A and C). With gene flow, the small number of congruent genealogies within these blocks have considerable positive asymmetry in external branch lengths (panel B) that is not found in the absence of migration (panel D). Farther from the *hetAB* mutation, congruent genealogies become common and their asymmetry in external branch lengths becomes negative, reflecting the difference in $N_{e}$ between the two populations. This recombination-dependent variation in branch length asymmetry is only seen when there is gene flow and is responsible for the nonmonotonic trend observed in Fig. 4c.

Overall, these simulations show that recombination can have a significant influence on our measure of genealogical asymmetry. Given that nonzero values of $A_{m}$ can be generated through recombination despite no post-divergence gene flow, we suggest that $A_{m}$ always be calculated across a range of block sizes and that the interaction between asymmetry and recombination be interpreted carefully. In the presence of recombination, evidence for post-divergence gene flow can be seen in values of $A_{m}$ that are stable and consistently nonzero across small block sizes (Fig. 4a). The pattern in Fig. 4c constitutes even stronger evidence for gene flow, as nonzero values of $A_{m}$ at small block sizes cannot be explained by recombination and differences in $N_{e}$, which instead decreases $A_{m}$ (as observed at large block sizes). This pattern is an expected consequence of a continent-island demographic history where the population with smaller $N_{e}$ receives gene flow forwards in time.

### Bidirectional gene flow

**Fig. 5.**
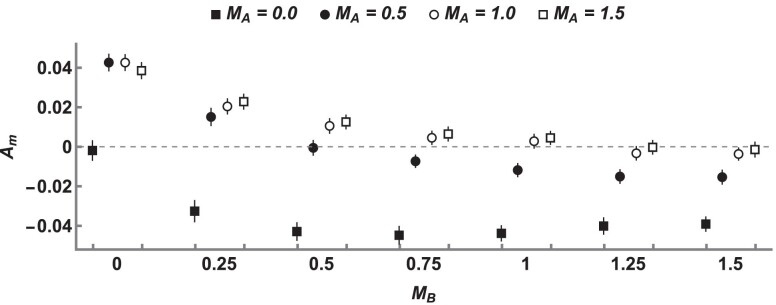
Expected values of $A_{m}$ obtained by coalescent simulation under an IM model with gene flow in both directions (500,000 replicates per-estimate). All simulations share the parameters $N_{A}=N_{B}=N_{AB}=100,000$, $\mu =10^{-8}$ per-site per-generation, and T=1.0. Simulations vary in the rate of migration from population *A* to population *B* (backwards in time) ($M_{A}$, denoted by color), as well as the rate of migration in the opposite direction ($M_{B}$, discrete *x*-axis). Error bars correspond to the 95% CIs of each estimate.

In the above sections, we have shown how undirectional gene flow generates asymmetry in the external branch lengths of incongruent genealogies. The assumption of strictly unidirectional gene flow allows exact derivations of branch lengths under the IM model using GFs. It is, however, more challenging to obtain analogous derivations for a model in which gene flow happens in both directions (see Lohse *et al*. 2016). We are nonetheless interested in whether asymmetry is generated under bidirectional gene flow. We therefore use coalescent simulations to estimate expectations for $A_{m}$ while allowing gene flow in both directions. We focus on the demography in Fig. 4a and vary the rate of gene flow in both directions, simulating short sequence blocks of 200 bases without recombination. Our simulations show that even a low rate of opposing gene flow can have a large effect on $A_{m}$ (Fig. 5). At the same time, we find that $A_{m}$ is typically nonzero under the demographic parameters we considered, and is only indistinguishable from zero when $M_{A}\approx M_{B}$. Additionally, $A_{m}$ tends to be positive when $M_{A}>M_{B}$ and negative when $M_{B}>M_{A}$ (Fig. 5), suggesting that $A_{m}$ provides information about the dominant direction of gene flow, at least in the case of similar effective population sizes. These simulation results also show that the total amount of gene flow (i.e. $M_{A}+M_{B}$) affects the magnitude of $A_{m}$. In particular as $M_{A}+M_{B}$ becomes large any difference between them is only weakly reflected in $A_{m}$. This mirrors the analytic results for unidirectional gene flow (Fig. 3d) because both are driven by the fact that very high migration rates lead to a coalescent process similar to that of a panmictic population. Overall, these results show that the power of $A_{m}$ to detect gene flow is reduced, but not removed, by bidirectional gene flow.

### Robustness to natural selection

Most methods for detecting past gene flow assume neutral evolution. Our approach also assumes a neutral coalescent process, yet we expect it to be robust to selective forces that increase the rate of coalescence within populations (e.g. selective sweeps) because congruent genealogies should not contribute to estimates of $A_{m}$. Other selective processes, such as balancing selection or background selection (BGS), can generate incongruent genealogies and so are more likely to influence estimates of $A_{m}$. To investigate whether $A_{m}$ is robust to this form of natural selection, we performed forward simulations of recombining genomes under a regime of BGS in which deleterious mutations have partially recessive fitness effects (see Methods for details).

We consider a four-taxon history with populations labeled [P1, P2, P3, O]. All populations experience BGS, and populations P1 and O are always strictly isolated from all others. We test for gene flow between P2 and P3 under three scenarios: (i) strict isolation between P2 and P3 (SI), (ii) SI with $N_{P3}$ twice the size of all others, and (iii) gene flow between P2 and P3 (see Methods for details). Using four populations allows us to compare the behavior of $A_{m}$ under BGS with the D-statistic of the ABBA-BABA test. As another comparison, we fit an IM model with bidirectional gene flow to the jSFS of P2 and P3 using moments (Jouganous *et al*. 2017). For a fair comparison, we used sequence polymorphism for all three analyses (rather than directly using simulated branch lengths for $A_{m}$ as above). For simplicity, we calculated $A_{m}$ for a single short block size (201 bases) rather than across a distribution.

**Fig. 6.**
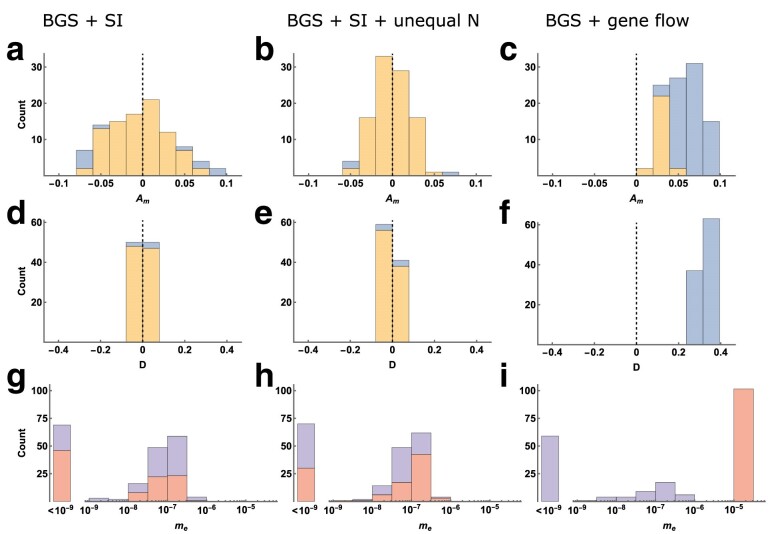
Estimates of $A_{m}$ (a, b, c), the D-statistic (d, e, f) and $m_{e}$ (g, h, i) from forward simulations with BGS (100 replicates per-scenario). Histograms on the left show results for a scenario of strict isolation (SI; (a, d, g)), whereas those in the middle show results for a SI scenario with unequal *N* between the focal populations (P2=10,000, P3=20,000; (b, e, h)), and those on the right show results for a scenario of undirectional gene flow from P3 to P2 forwards in time ($m_{P2}=2.5\times 10^{-5}$, $M_{P2}=0.5$; (c, f, i)). Histogram bars showing $A_{m}$ and *D* are colored blue when values are significantly greater or less than zero (p<0.05), and yellow otherwise. Histograms of $m_{e}$ estimates from the jSFS have a $log_{10}$*x*-axis, with a single-point mass for values $<10^{-9}$. Histogram bars are colored red for estimates of $m_{P2}$ and purple for estimates of $m_{P3}$.

First, simulating a history of strict isolation with BGS (Figure 6a, d, g) results in estimates of $A_{m}$ that are centered around zero. Only a small minority of estimates are significantly greater or less than zero (false positive rate (FPR) = 0.11). The same is true for the D-statistic (FPR = 0.05), but estimates of $m_{e}$ from the jSFS are often nonzero ($m_{P2}+m_{P3}>10^{-9}$ in 89% of replicates), broadly consistent with the results of Smith and Hahn (2024). Second, when P3 is twice the size of all other populations (Fig. 6b, e, h), we find that estimates of $A_{m}$ and *D* are again centred around zero, with low false positive rates (0.03 and 0.06 respectively). Estimates of $m_{e}$ from the jSFS are again low, but nonzero in most cases. Lastly, when we included unidirectional gene flow from P3 to P2 (forwards in time, $m_{P2}=2.5\times 10^{-5}$, $M_{P2}=0.5$; Fig. 6c, f, i), all three approaches provide strong evidence for gene flow. The D-statistic detects gene flow between P2 and P3 in every simulation replicate, although this result does require that the analysis is conditioned on the true species tree topology. Similarly, estimates of $A_{m}$ are always positive and are significantly greater than zero in most cases (true positive rate = 0.74). Estimates of $m_{P2}$ from the jSFS (i.e. the rate corresponding to the simulated unidirectional gene flow) are very accurate (lower quartile, median and upper quartile across replicates of 2.52,2.63, and $2.75\times 10^{-5}$).

### Detecting gene flow between *Heliconius* butterflies

**Fig. 7.**
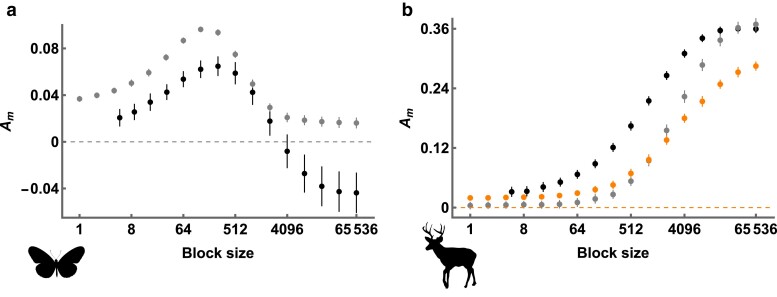
Distributions of $A_{m}$ (*y*-axis) across block sizes (*x*-axis, log2 scale) for a) *Heliconius melpomene* and *H. cydno*, and b) *Odocoileus virginianus* and *O. hemionus*. In both panels, black points correspond to values of $A_{m}$ estimated directly from WGS data. Gray points in (a) correspond to values of $A_{m}$ expected under the demographic history inferred by Laetsch *et al*. (2023). Gray points in (b) correspond to values of $A_{m}$ expected under the demographic history inferred by Kessler and Shafer (2024) and presented in their Fig. 4a. Orange points correspond to values for the alternative history presented in Fig. 4b of Kessler and Shafer (2024). Error bars correspond to the 95% CIs of each estimate.

To demonstrate how genealogical asymmetry can be used to detect historical gene flow between two populations, we re-analyze genome sequence data from a pair of closely related butterfly species: *Heliconius melpomene* and *H. cydno*. These species exhibit strong assortative mating, and the rare natural hybrids are fertile only when male. Despite this, previous analyses of genome sequence data have revealed evidence for post-divergence gene flow from *H. cydno* to *H. melpomene* forwards in time (Kronforst *et al*. 2013; Martin *et al*. 2013; Lohse *et al*. 2016; Laetsch *et al*. 2023). We calculated $A_{m}$ across a range of block sizes for a pair of male individuals (one sampled from each species and both from Panama). At short block sizes, $A_{m}$ is positive (Fig. 7a). As the block size increases, however, $A_{m}$ eventually decreases and reaches negative values (Fig. 7a). This matches the pattern observed in Fig. 4c and constitutes strong evidence for gene flow from *H. cydno* to *H. melpomene*.

We can also compare our $A_{m}$ values to those expected under the demographic history of *H. melpomene* and *H. cydno* as inferred by Laetsch *et al*. (2023), whose data we reanalyzed. Interestingly, simulating under the inferred demography generates $A_{m}$ values that only weakly match those from the data (Fig. 7a). This is somewhat surprising given that Laetsch *et al*. (2023) fit their model to blockwise data that is informative about the joint distribution of genealogical branch lengths, and therefore contains information about $A_{m}$ (see Discussion). The discrepancy could be because the approach of Laetsch *et al*. (2023) assumes no recombination within short sequence blocks, as well as an IM demography with unidirectional gene flow. Although our analytic results have the same assumptions, summarizing $A_{m}$ across a range of block sizes does not require any assumptions about recombination or demography.

### Detecting gene flow between *Odocoileus* deer

We expect our approach for detecting gene flow to perform particularly well for the pair of *Heliconius* species analyzed above, as the long-term rate of gene flow is intermediate (M≈1) and the smaller $N_{e}$ population receives migrants forwards in time (Kronforst *et al*. 2013; Lohse *et al*. 2016). Using the same approach to detect low levels of bidirectional gene flow between sister taxa is likely more challenging. As an example, we re-analyze genome sequence data from white-tailed and mule deer: *Odocoileus virginianus* and *O. hemionus*. Although hybridization is observed between present-day populations, Kessler *et al*. (2023) showed that the distribution of coalescence times within and between these species (inferred with MSMC-IM; Wang *et al*. 2020) is consistent with a history of speciation without gene flow. The same authors recently expanded their dataset and fit demographic models to the jSFS with dadi (Gutenkunst *et al*. 2009), inferring low levels of gene flow via secondary contact between the two species (Kessler and Shafer 2024). We calculated $A_{m}$ across a range of block sizes for a pair of individuals with high coverage genome sequences data (Ov_ON6 and Oh_WA1). We find that $A_{m}$ is positive when blocks are short and monotonically increases with block length (Fig. 7b). Because we cannot estimate $A_{m}$ in the complete absence of recombination, it is difficult to tell whether these results are evidence of a demographic history with post-divergence gene flow (Fig. 4a), or a history without gene flow but a large difference in $N_{e}$ between the two populations (Fig. 4b).

We next perform simulations under two alternative demographic histories inferred by Kessler and Shafer (2024). The first of these demographies includes a low rate of bidirectional secondary contact gene flow, where the probability that a single lineage migrates at least once is low (P=0.015). Expected $A_{m}$ values for this demography are indistinguishable from zero at small block sizes (grey points in Fig. 7b; $A_{m}=0.004\;(\;-0.002,0.010)$ for a block size of 1 base). The second demography includes bidirectional gene flow between the present and the onset of divergence and lineages have a greater probability of migration (P=0.044). In this case, simulated $A_{m}$ values are greater than zero across all block sizes (orange points in Fig. 7b; $A_{m}=0.016\;(0.009,0.023)$ for a block size of 1 base). We find that $A_{m}$ values estimated directly from the data are greater than those of both simulated demographies at small block sizes (Fig. 7b). This simulation check therefore suggests that there is indeed some evidence for historic gene flow from *O. hemionus* to *O. virginianus*, forwards in time, even if the signal is subtle overall.

## Discussion

Understanding how past demography shapes patterns of genome sequence variation is a major goal of evolutionary genetics. In this work, we have shown that gene flow between two recently diverged populations leads to an asymmetry in the length of external branches on genealogies that are incongruent with the species history. This asymmetry arises when (i) lineages that were sampled from the same population become trapped in different populations by a single migration event, (ii) the final coalescent event involves the isolated lineage, and (iii) this coalescence uniquely occurs only after a second migration or a merging of populations (Fig. 2a). Importantly, we have shown that this effect is identifiable from WGS data, and we used this to define an asymmetry-based test for gene flow that requires data from only two taxa.

### Comparison with other approaches

Our approach to detecting gene flow shares several similarities with the ABBA-BABA test (Green *et al*. 2010; Durand *et al*. 2011). Both focus on incongruent genealogies, and both rely on the expectation that particular branches are (on average) equal in length under a history of strict isolation but unequal under a history of gene flow. They also both share the general strengths of asymmetry-based methods: they require few assumptions about the demographic history and are expected to be robust to the effects of natural selection (Fig. 6). There are, however, several important differences between the two approaches. For one, $A_{m}$ provides information about the direction of gene flow (Fig. 5), whereas the ABBA-BABA test does not (although see Pease and Hahn 2015 and Leppälä *et al*. 2024 for five-taxon extensions that do). Additionally, the ABBA-BABA test statistic (D) typically scales with the rate of gene flow, whereas $A_{m}$ has a nonlinear and nonmonotone relationship with *M*, making detection of gene flow at very high rates challenging (Fig. 3). Another key difference is that the ABBA-BABA test assumes independence among all sites while $A_{m}$ uses information from linked polymorphisms. As we do not know the true span of any genealogy, we can only sample polymorphisms from small regions that we (optimistically) assume are nonrecombining. Additionally, the requirement of linkage information means that estimation of $A_{m}$ will typically require WGS data (rather than RNA-seq or RAD-seq), especially in species with low genetic diversity. So although $A_{m}$ shares many of the strengths of other asymmetry-based statistics, the requirement of analyzing linked polymorphisms with occasional recombination means that estimates of $A_{m}$ are not always as straightforward to obtain or interpret (see below). At the same time, $A_{m}$ does have the major advantage of only requiring samples from two-taxa, rather than four.

Like most asymmetry-based methods, our approach focuses on a very specific signal in WGS data to detect past gene flow. By contrast, model-based methods tend to use more comprehensive summaries of WGS data to infer past demography. Do model-based methods then implicitly use asymmetry in external branch lengths to detect gene flow? Several popular demographic inference methods (Gutenkunst *et al*. 2009; Jouganous *et al*. 2017; Kamm *et al*. 2020; Excoffier *et al*. 2021) use the jSFS as a convenient way to summarize polymorphism data from two or more populations. Because the jSFS only contains information about average branch lengths, it does not preserve direct information about asymmetry in branch lengths on incongruent genealogies. Instead, the signal of gene flow in the jSFS is mostly contained in the sharing of low frequency derived alleles between populations (Gutenkunst *et al*. 2009), therefore requiring a large sample size.

Other demographic inference methods use information from the joint distribution of branch lengths (Gronau *et al*. 2011; Flouri *et al*. 2023; Laetsch *et al*. 2023) and we therefore do expect these methods to implicitly use $A_{m}$ when inferring rates of gene flow. Interestingly, we found that the distribution of $A_{m}$ values for the pair of *Heliconius* species did not match those expected under the demography inferred by Laetsch *et al*. (2023) (Fig. 7a). This could be because their inference approach (which also uses the GF calculation from Lohse *et al*. 2011, 2016) assumes no recombination within a 64 bp sequence block (note that the expected value of $A_{m}$ for a block size of 1 bp under their demography is similar to observed $A_{m}$ for a 64 bp block in the data; Fig. 7a). Alternatively, the difference could be because their method considers more than just incongruent asymmetry to infer gene flow. For example, the density of *fixed* polymorphisms provides information for inference under the approach of Laetsch *et al*. (2023), but is absent from $A_{m}$.

### The recombination problem

One strength of our approach is that we have an explicit expectation for $A_{m}$ under a history of strict isolation ($A_{m}=0$), whereas many related summary statistics instead require coalescent simulations to obtain expected values (Geneva *et al*. 2015; Rosenzweig *et al*. 2016; Hibbins and Hahn 2022). However, our analytic results only hold in the absence of recombination (Fig. 4). Indeed, like most inference approaches in population genetics, the power of the $A_{m}$ statistic depends on the relative rates of recombination *ρ* and mutation *θ* (Supplementary Fig. 2). The higher the recombination rate, the faster $A_{m}$ departs from our analytic predictions. In contrast, the higher the mutation rate, the greater the information to accurately estimate $A_{m}$ for small block sizes where the effect of recombination is negligible. The power of $A_{m}$ is therefore greatest when ρ/θ is small (Supplementary Fig. 2).

To some extent, we have mitigated the effect of recombination by calculating $A_{m}$ across varying block sizes, as some distributions provide unambiguous evidence for gene flow (Figs. 4c and 7a). Our reanalysis of WGS data from *Odocoileus* deer, however, shows that certain demographic histories make detection of gene flow with $A_{m}$ challenging (Fig. 7b). More specifically, when the difference in $N_{e}$ between populations is large, congruent genealogies tend to have highly asymmetrical external branch lengths (Fig. 1c). As a result, blocks that span congruent genealogies due to hidden recombination events will have nonzero values of $A_{m}$, even in the absence of migration. In the case of the *Odocoileus* deer, it was helpful to perform coalescent simulations of previously inferred demographic histories (Kessler and Shafer 2024). These simulations suggested that $A_{m}$ is likely nonzero due to past gene flow, rather than due to differences in $N_{e}$ alone, although this interpretation does assume that the simulated recombination rate is similar to the true rate. Because of these issues, the influence of recombination on estimates of $A_{m}$ is certainly a weakness of our approach, especially when ρ/θ is large.

There are, in principle, several ways to remove the influence of recombination from estimates of $A_{m}$. One option is to calculate $A_{m}$ (or $A_{i}$) from the marginal genealogies of a reconstructed ARG (Rasmussen *et al*. 2014; Kelleher *et al*. 2019; Speidel *et al*. 2019). A perfectly inferred ARG would provide unbiased estimates of $A_{m}$, although it is already possible to obtain much more detailed information about gene flow from such data (Wang *et al*. 2020; Pope *et al*. 2023). An alternative strategy is to derive expectations for $A_{m}$ in the presence of low rates of recombination, for example, by including recombination in the GF (Lohse *et al*. 2011). Note, however, that analytic expectations would depend on all five parameters of the IM model as well as the rate of recombination. This means that estimation of $A_{m}$ would become a challenging model-based inference problem. There does not seem to be any way to easily adjust $A_{m}$ to be robust to recombination, and so careful interpretation of $A_{m}$ distributions (Figs. 4 and 7) may be the most straightforward way to deal with this issue.

### Misspecified models

The approach for detecting gene flow that we have presented here does not require the specification of any particular model, and so should not suffer from model mis-specification issues in the same way as other demographic inference methods (Becquet and Przeworski 2009; Momigliano *et al*. 2021). The approach is nonetheless inspired by properties of incongruent genealogies under the IM model, so the population history does need to at least partially resemble an IM model for the test to be valid. For instance, $A_{m}$ should have similar properties for models of secondary contact or ancient migration. In particular, the expectation of $A_{m}=0$ under strict isolation will hold for these models because they are equivalent to the IM model when $m_{e}=0$. More significant departures from the IM model can, however, lead to misinference. While $A_{m}$ is robust to variation in mutation rates *along the genome*, the statistic is sensitive to variation in mutation rates *among populations*. Throughout we have assumed that mutation rates are the same among populations so that the number of mutations observed is directly proportional to the underlying branch lengths. This will not be the case if mutation rates differ between populations. Although it is difficult under the full model, for a strict isolation model it is straightforward to write an expression for $A_{i}$ (in terms of mutation counts) while allowing population-specific mutation rates:


$$A_{i}=\frac{3t(\mu_{A}-\mu_{B})}{8N_{AB}\mu_{AB}+3t(\mu_{A}-\mu_{B})}.$$

If $\mu_{A}=\mu_{B}$, then $A_{i}=0$ as expected. However if $\mu_{A}\neq \mu_{B}$, $A_{i}$ is nonzero: for example, if $\mu_{AB}=\mu_{B}$, $t=2N_{AB}$, and $\mu_{A}=2\mu_{B}$, then $A_{i}=0.231$. Variation in mutation rate between populations can therefore lead to considerable biases in estimates of $A_{m}$.

Even if mutation rates are shared between populations, the IM model still focuses on just two populations and ignores potentially complex histories of gene flow from other sources. Introgression from an un-sampled ‘ghost’ population has been shown to affect the results of the ABBA-BABA test (Tricou *et al*. 2022). We expect a similar effect on $A_{m}$; if *A* and *B* are strictly isolated populations but *A* experiences gene flow from an outgroup ghost population, then $A_{m}$ is likely to give a false positive signal of gene flow from *B* to *A* (forwards in time). However, if population *A* and the ghost population share a common ancestor more recently than populations *A* and *B*, then $A_{m}$ will be unbiased by ghost gene flow into *A*. This is because any (uncoalesced) lineages originally sampled from *A* will still be exchangeable once they reach the ancestral (*AB*) population, even if one of them spent time in the ghost population (i.e. equation 2 will still hold). Similar logic applies to other demographic events that affect a single population; as long as all four lineages enter the ancestral population at the same time and are exchangeable, then $A_{m}=0$ under strict isolation. So while the utility of the $A_{m}$ statistic does depend on certain model assumptions, it is also robust to demographic events that model-based methods may not be.

### Outlook

In this work, we have shown that gene flow between two recently diverged populations leads to an identifiable asymmetry in genealogical branch lengths. Much of our focus has been on defining and exploring the properties of an asymmetry-based two-taxon test for gene flow. We do not, however, suggest that our test take the place of current model-based inference methods. Instead, we anticipate that the test will complement existing approaches, as it focuses on a particular signal that model-based methods either fail to capture or conflate with other information. Indeed, $A_{m}$ may be an informative and complementary summary statistic for model-based inference using an approximate Bayesian computation approach. More generally, we hope that our results provide some useful intuition about how gene flow shapes genealogical histories and genome sequence variation.

## Supplementary Material

iyae157_Supplementary_Data

## Data Availability

Mathematica and python notebooks can be accessed at https://github.com/A-J-F-Mackintosh/two_taxon_asymmetry. The Mathematica notebook is also available as a Supplementary pdf file (File 1). The python script estimate_Am.py can be accessed at the same github repository (GNU General Public License v3.0). Sequence data from Laetsch *et al*. (2023) is available at the NCBI Sequence Read Archive (Bioprojects PRJEB11772 and PRJEB1749), as is the data associated with Kessler *et al*. (2023) (BioProject PRJNA830519). Supplemental material available at GENETICS online.

## References

[iyae157-B1] Baumdicker F , Bisschop G, Goldstein D, Gower G, Ragsdale AP, Tsambos G, Zhu S, Eldon B, Ellerman EC, Galloway JG, *et al*. 2021. Efficient ancestry and mutation simulation with msprime 1.0. Genetics. 220(3):iyab229. doi:10.1093/genetics/iyab229 PMC9176297 34897427

[iyae157-B2] Becquet C , Przeworski M. 2009. Learning about modes of speciation by computational approaches. Evolution. 63(10):2547–2562. doi:10.1111/evo.2009.63.issue-10 19228187

[iyae157-B3] Beerli P , Felsenstein J. 1999. Maximum-likelihood estimation of migration rates and effective population numbers in two populations using a coalescent approach. Genetics. 152(2):763–773. doi:10.1093/genetics/152.2.763 10353916 PMC1460627

[iyae157-B4] Chen L , Qiu Q, Jiang Y, Wang K, Lin Z, Li Z, Bibi F, Yang Y, Wang J, Nie W, *et al*. 2019. Large-scale ruminant genome sequencing provides insights into their evolution and distinct traits. Science. 364(6446):eaav6202. doi:10.1126/science.aav6202 31221828

[iyae157-B5] Cousins T , Scally A, Durbin R. 2024. A structured coalescent model reveals deep ancestral structure shared by all modern humans. bioRxiv. pp. 2024–03

[iyae157-B6] Davey JW , Barker SL, Rastas PM, Pinharanda A, Martin SH, Durbin R, McMillan WO, Merrill RM, Jiggins CD. 2017. No evidence for maintenance of a sympatric *Heliconius* species barrier by chromosomal inversions. Evol Lett. 1:138–154. doi:10.1002/evl3.12 30283645 PMC6122123

[iyae157-B7] Durand EY , Patterson N, Reich D, Slatkin M. 2011. Testing for ancient admixture between closely related populations. Mol Biol Evol. 28:2239–2252. doi:10.1093/molbev/msr048 21325092 PMC3144383

[iyae157-B8] Eaton DA , Ree RH. 2013. Inferring phylogeny and introgression using RADseq data: an example from flowering plants (Pedicularis: Orobanchaceae). Syst Biol. 62:689–706. doi:10.1093/sysbio/syt032 23652346 PMC3739883

[iyae157-B9] Excoffier L , Marchi N, Marques DA, Matthey-Doret R, Gouy A, Sousa VC. 2021. fastsimcoal2: demographic inference under complex evolutionary scenarios. Bioinformatics. 37:4882–4885. doi:10.1093/bioinformatics/btab468 34164653 PMC8665742

[iyae157-B10] Flouri T , Jiao X, Huang J, Rannala B, Yang Z. 2023. Efficient Bayesian inference under the multispecies coalescent with migration. Proc Natl Acad Sci USA. 120(44):e2310708120. doi:10.1073/pnas.2310708120 37871206 PMC10622872

[iyae157-B11] Flouri T , Jiao X, Rannala B, Yang Z. 2020. A Bayesian implementation of the multispecies coalescent model with introgression for phylogenomic analysis. Mol Biol Evol. 37:1211–1223. doi:10.1093/molbev/msz296 31825513 PMC7086182

[iyae157-B12] Frankel LE , Ané C. 2023. Summary tests of introgression are highly sensitive to rate variation across lineages. Syst Biol. 72(6):1357–1369. doi:10.1093/sysbio/syad056 37698548

[iyae157-B13] Galtier N . 2024. An approximate likelihood method reveals ancient gene flow between human, chimpanzee and gorilla. Peer Comm J. 4:e3. doi:10.24072/pcjournal.359

[iyae157-B14] Geneva AJ , Muirhead CA, Kingan SB, Garrigan D. 2015. A new method to scan genomes for introgression in a secondary contact model. PLoS One. 10(4):e0118621. doi:10.1371/journal.pone.0118621 25874895 PMC4396994

[iyae157-B15] Green RE , Krause J, Briggs AW, Maricic T, Stenzel U, Kircher M, Patterson N, Li H, Zhai W, Fritz MHY, *et al*. 2010. A draft sequence of the Neandertal genome. Science. 328(5979):710–722. doi:10.1126/science.1188021 20448178 PMC5100745

[iyae157-B16] Gronau I , Hubisz MJ, Gulko B, Danko CG, Siepel A. 2011. Bayesian inference of ancient human demography from individual genome sequences. Nat Genet. 43(10):1031–1034. doi:10.1038/ng.937 21926973 PMC3245873

[iyae157-B17] Gutenkunst RN , Hernandez RD, Williamson SH, Bustamante CD. 2009. Inferring the joint demographic history of multiple populations from multidimensional SNP frequency data. PLoS Genet. 5(10):e1000695. doi:10.1371/journal.pgen.1000695 19851460 PMC2760211

[iyae157-B18] Haller BC , Galloway J, Kelleher J, Messer PW, Ralph PL. 2019. Tree-sequence recording in SLiM opens new horizons for forward-time simulation of whole genomes. Mol Ecol Res. 19:552–566. doi:10.1111/men.2019.19.issue-2 PMC6393187 30565882

[iyae157-B19] Haller BC , Messer PW. 2023. SLiM 4: multispecies eco-evolutionary modeling. Am Nat. 201:E127–E139. doi:10.1086/723601 37130229 PMC10793872

[iyae157-B20] Hamlin JA , Hibbins MS, Moyle LC. 2020. Assessing biological factors affecting postspeciation introgression. Evol Lett. 4:137–154. doi:10.1002/evl3.159 32313689 PMC7156103

[iyae157-B21] Hey J , Nielsen R. 2004. Multilocus methods for estimating population sizes, migration rates and divergence time, with applications to the divergence of *Drosophila pseudoobscura* and *D. persimilis*. Genetics. 167:747–760. doi:10.1534/genetics.103.024182 15238526 PMC1470901

[iyae157-B22] Hibbins MS , Hahn MW. 2022. Phylogenomic approaches to detecting and characterizing introgression. Genetics. 220:iyab173. doi:10.1093/genetics/iyab173 34788444 PMC9208645

[iyae157-B23] Huang J , Thawornwattana Y, Flouri T, Mallet J, Yang Z. 2022. Inference of gene flow between species under misspecified models. Mol Biol Evol. 39:msac237. doi:10.1093/molbev/msac237 36317198 PMC9729068

[iyae157-B24] Jensen A , Swift F, de Vries D, Beck RM, Kuderna LF, Knauf S, Chuma IS, Keyyu JD, Kitchener AC, Farh K, *et al*. 2023. Complex evolutionary history with extensive ancestral gene flow in an African primate radiation. Mol Biol Evol. 40:msad247. doi:10.1093/molbev/msad247 37987553 PMC10691879

[iyae157-B25] Jiao X , Flouri T, Yang Z. 2021. Multispecies coalescent and its applications to infer species phylogenies and cross-species gene flow. Nat Sci Rev. 8:nwab127. doi:10.1093/nsr/nwab127 PMC8692950 34987842

[iyae157-B26] Johnston SE , Huisman J, Ellis PA, Pemberton JM. 2017. A high-density linkage map reveals sexual dimorphism in recombination landscapes in red deer (*Cervus elaphus*). G3 Gen Genom Genet. 7:2859–2870. doi:10.1534/g3.117.044198 PMC5555489 28667018

[iyae157-B27] Jouganous J , Long W, Ragsdale AP, Gravel S. 2017. Inferring the joint demographic history of multiple populations: beyond the diffusion approximation. Genetics. 206:1549–1567. doi:10.1534/genetics.117.200493 28495960 PMC5500150

[iyae157-B28] Kamm J , Terhorst J, Durbin R, Song YS. 2020. Efficiently inferring the demographic history of many populations with allele count data. J Am Stat Assoc. 115:1472–1487. doi:10.1080/01621459.2019.1635482 33012903 PMC7531012

[iyae157-B29] Keightley PD , Pinharanda A, Ness RW, Simpson F, Dasmahapatra KK, Mallet J, Davey JW, Jiggins CD. 2015. Estimation of the spontaneous mutation rate in *Heliconius melpomene*. Mol Biol Evol. 32:239–243. doi:10.1093/molbev/msu302 25371432 PMC4271535

[iyae157-B30] Kelleher J , Wong Y, Wohns AW, Fadil C, Albers PK, McVean G. 2019. Inferring whole-genome histories in large population datasets. Nat Genet. 51:1330–1338. doi:10.1038/s41588-019-0483-y 31477934 PMC6726478

[iyae157-B31] Kessler C , Shafer ABA. 2024. Genomic analyses capture the human-induced demographic collapse and recovery in wide-ranging cervid. Mol Biol Evol. 41:msae038. doi:10.1093/molbev/msae038 38378172 PMC10917209

[iyae157-B32] Kessler C , Wootton E, Shafer ABA. 2023. Speciation without gene-flow in hybridizing deer. Mol Ecol. 32:1117–1132. doi:10.1111/mec.v32.5 36516402

[iyae157-B33] Kingman JF . 1982. On the genealogy of large populations. J Appl Prob. 19:27–43. doi:10.2307/3213548

[iyae157-B34] Koppetsch T , Malinsky M, Matschiner M. 2024. Towards reliable detection of introgression in the presence of among-species rate variation. Syst Biol. syae028. doi:10.1093/sysbio/syae028 PMC11639170 38912803

[iyae157-B35] Kronforst MR , Hansen ME, Crawford NG, Gallant JR, Zhang W, Kulathinal RJ, Kapan DD, Mullen SP. 2013. Hybridization reveals the evolving genomic architecture of speciation. Cell Rep. 5:666–677. doi:10.1016/j.celrep.2013.09.042 24183670 PMC4388300

[iyae157-B36] Kulathinal RJ , Stevison LS, Noor MA. 2009. The genomics of speciation in *Drosophila*: diversity, divergence, and introgression estimated using low-coverage genome sequencing. PLoS Genet. 5:e1000550. doi:10.1371/journal.pgen.1000550 19578407 PMC2696600

[iyae157-B37] Laetsch DR , Bisschop G, Martin SH, Aeschbacher S, Setter D, Lohse K. 2023. Demographically explicit scans for barriers to gene flow using gIMble. PLoS Genet. 19:e1010999. doi:10.1371/journal.pgen.1010999 37816069 PMC10610087

[iyae157-B38] Leppälä K , da Silva Coelho FA, Richter M, Albert VA, Lindqvist C. 2024. Five-leaf generalizations of the D-statistic reveal the directionality of admixture. Mol Biol Evol. msae198. doi:10.1093/molbev/msae198 39302159 PMC11708231

[iyae157-B39] Lohse K , Chmelik M, Martin SH, Barton NH. 2016. Efficient strategies for calculating blockwise likelihoods under the coalescent. Genetics. 202:775–786. doi:10.1534/genetics.115.183814 26715666 PMC4788249

[iyae157-B40] Lohse K , Frantz LA. 2014. Neandertal admixture in eurasia confirmed by maximum-likelihood analysis of three genomes. Genetics. 196:1241–1251. doi:10.1534/genetics.114.162396 24532731 PMC3982695

[iyae157-B41] Lohse K , Harrison RJ, Barton NH. 2011. A general method for calculating likelihoods under the coalescent process. Genetics. 189:977–987. doi:10.1534/genetics.111.129569 21900266 PMC3213358

[iyae157-B42] Lopez Fang L , Peede D, Ortega-Del Vecchyo D, McTavish EJ, Huerta-Sanchez E. 2024. Leveraging shared ancestral variation to detect local introgression. PLoS Genet. 20:e1010155. doi:10.1371/journal.pgen.1010155 38190420 PMC10798638

[iyae157-B43] Malinsky M , Challis RJ, Tyers AM, Schiffels S, Terai Y, Ngatunga BP, Miska EA, Durbin R, Genner MJ, Turner GF. 2015. Genomic islands of speciation separate cichlid ecomorphs in an East African crater lake. Science. 350:1493–1498. doi:10.1126/science.aac9927 26680190 PMC4700518

[iyae157-B44] Malinsky M , Matschiner M, Svardal H. 2021. Dsuite - Fast D-statistics and related admixture evidence from VCF files. Mol Ecol Res. 21:584–595. doi:10.1111/men.v21.2 PMC7116594 33012121

[iyae157-B45] Marcionetti A , Bertrand JA, Cortesi F, Donati GF, Heim S, Huyghe F, Kochzius M, Pellissier L, Salamin N. 2024. Recurrent gene flow events occurred during the diversification of clownfishes of the skunk complex. Mol Ecol. 33(11):e17347. doi:10.1111/mec.v33.11 38624248

[iyae157-B46] Martin SH , Amos W. 2021. Signatures of introgression across the allele frequency spectrum. Mol Biol Evol. 38:716–726. doi:10.1093/molbev/msaa239 32941617 PMC7826190

[iyae157-B47] Martin SH , Dasmahapatra KK, Nadeau NJ, Salazar C, Walters JR, Simpson F, Blaxter M, Manica A, Mallet J, Jiggins CD. 2013. Genome-wide evidence for speciation with gene flow in *Heliconius* butterflies. Genome Res. 23:1817–1828. doi:10.1101/gr.159426.113 24045163 PMC3814882

[iyae157-B48] Martin SH , Davey JW, Jiggins CD. 2015. Evaluating the use of ABBA–BABA statistics to locate introgressed loci. Mol Biol Evol. 32:244–257. doi:10.1093/molbev/msu269 25246699 PMC4271521

[iyae157-B49] Momigliano P , Florin AB, Merilä J. 2021. Biases in demographic modeling affect our understanding of recent divergence. Mol Biol Evol. 38:2967–2985. doi:10.1093/molbev/msab047 33624816 PMC8233503

[iyae157-B50] Pease JB , Hahn MW. 2015. Detection and polarization of introgression in a five-taxon phylogeny. Syst Biol. 64:651–662. doi:10.1093/sysbio/syv023 25888025

[iyae157-B51] Pope NS , Singh A, Childers AK, Kapheim KM, Evans JD, López-Uribe MM. 2023. The expansion of agriculture has shaped the recent evolutionary history of a specialized squash pollinator. Proc Natl Acad Sci USA. 120:e2208116120. doi:10.1073/pnas.2208116120 37011184 PMC10104555

[iyae157-B52] Quenouille MH . 1949. Approximate tests of correlation in time-series 3. In:. volume 45. pp. 483–484. Cambridge University Press.

[iyae157-B53] Rannala B , Yang Z. 2003. Bayes estimation of species divergence times and ancestral population sizes using DNA sequences from multiple loci. Genetics. 164(4):1645–1656. doi:10.1093/genetics/164.4.1645 12930768 PMC1462670

[iyae157-B54] Rasmussen MD , Hubisz MJ, Gronau I, Siepel A. 2014. Genome-wide inference of ancestral recombination graphs. PLoS Genet. 10:e1004342. doi:10.1371/journal.pgen.1004342 24831947 PMC4022496

[iyae157-B55] Rogers AR . 2019. Legofit: estimating population history from genetic data. BMC Bioinformatics. 20:1–10. doi:10.1186/s12859-019-3154-1 31660852 PMC6819480

[iyae157-B56] Rosenzweig BK , Pease JB, Besansky NJ, Hahn MW. 2016. Powerful methods for detecting introgressed regions from population genomic data. Mol Ecol. 25:2387–2397. doi:10.1111/mec.2016.25.issue-11 26945783 PMC4899106

[iyae157-B57] Satokangas I , Nouhaud P, Seifert B, Punttila P, Schultz R, Jones M, Siren J, Helanterä H, Kulmuni J. 2023. Semipermeable species boundaries create opportunities for gene flow and adaptive potential. Mol Ecol. 32:4329–4347. doi:10.1111/mec.v32.15 37222024

[iyae157-B58] Shafer ABA , Peart CR, Tusso S, Maayan I, Brelsford A, Wheat CW, Wolf JB. 2017. Bioinformatic processing of RAD-seq data dramatically impacts downstream population genetic inference. Methods Ecol Evol. 8:907–917. doi:10.1111/mee3.2017.8.issue-8

[iyae157-B59] Slatkin M , Pollack JL. 2008. Subdivision in an ancestral species creates asymmetry in gene trees. Mol Biol Evol. 25:2241–2246. doi:10.1093/molbev/msn172 18689871 PMC2734134

[iyae157-B60] Smith ML , Hahn MW. 2024. Selection leads to false inferences of introgression using popular methods. Genetics. 227(4):iyae089. doi:10.1093/genetics/iyae089 38805070

[iyae157-B61] Speidel L , Forest M, Shi S, Myers SR. 2019. A method for genome-wide genealogy estimation for thousands of samples. Nat Genet. 51(9):1321–1329. doi:10.1038/s41588-019-0484-x 31477933 PMC7610517

[iyae157-B62] Tajima F . 1983. Evolutionary relationship of DNA sequences in finite populations. Genetics. 105:437–460. doi:10.1093/genetics/105.2.437 6628982 PMC1202167

[iyae157-B63] Takahata N . 1989. Gene genealogy in three related populations: consistency probability between gene and population trees. Genetics. 122:957–966. doi:10.1093/genetics/122.4.957 2759432 PMC1203770

[iyae157-B64] Takahata N , Satta Y, Klein J. 1995. Divergence time and population size in the lineage leading to modern humans. Theor Popul Biol. 48:198–221. doi:10.1006/tpbi.1995.1026 7482371

[iyae157-B65] Tricou T , Tannier E, de Vienne DM. 2022. Ghost lineages highly influence the interpretation of introgression tests. Syst Biol. 71:1147–1158. doi:10.1093/sysbio/syac011 35169846 PMC9366450

[iyae157-B66] Wang K , Mathieson I, O’Connell J, Schiffels S. 2020. Tracking human population structure through time from whole genome sequences. PLoS Genet. 16:e1008552. doi:10.1371/journal.pgen.1008552 32150539 PMC7082067

[iyae157-B67] Xiong T , Li X, Yago M, Mallet J. 2022. Admixture of evolutionary rates across a butterfly hybrid zone. Elife. 11:e78135. doi:10.7554/eLife.78135 35703474 PMC9246367

