# Supplementary material for: Genealogical asymmetry under the isolation with migration model and a two-taxon test for gene flow
Source: Genetics. 2024 Sep 30;228(4):iyae157. doi: 10.1093/genetics/iyae157 (PMC11631468; doi:10.1093/genetics/iyae157)

Functions to make GF for the IM model - adapted from Lohse et al 2016

In[19]:=

```
testState = {{}, {{a}, {a}}, {{b}, {b}}}
```

Out[19]=

```
{{}, {{a}, {a}}, {{b}, {b}}}
```

In[20]:=

```
TakeAway::usage =
  "TakeAway[U,V]  gives the set U, minus elements V. Note that this is not
    the same as Complement[U,V] if U contains repeated elements. ";
```

In[21]:=

```
TakeAway[U_List, {}] := U;
(*TakeAway[U_List,{{}}]:=If[MemberQ[U,{{}}],TakeAway[U,{{}}],U];*)
TakeAway[U_List, {v_}] :=
  Drop[U, Cases[Position[U, v], {_Integer}][[1]]] /; Complement[{v}, U] == {};
TakeAway[U_List, V_List] :=
  TakeAway[TakeAway[U, Take[V, 1]], Drop[V, 1]] /; Complement[V, U] == {};
TakeAway[U_List, W__List, V_List] := TakeAway[TakeAway[U, W], V];
```

In[25]:=

```
GetVars[expr_, gfTerm_] := Cases[Variables[gfTerm], expr[_]]
```

In[26]:=

```
MakeCoalEvents[pL_List] := Module[
  {lambda , pIndex = {1, 2, 3}, pRates = {λab, λa, λb},
   coals = Tally[Subsets[Sort[#], {2}]] & /@ pL, ppL, idx1, pair, rate, res},
  coals =
   MapThread[#1 //. {y_List, x_Integer} :> {y, x * #2, #3} &, {coals, pRates, pIndex}];
  coals = coals~Flatten~1;
  res = {};
  For[i = 1, i ≤ Length[coals], i++,
   pair = coals[[i]][[1]];
   rate = coals[[i]][[2]];
   idx1 = coals[[i]][[3]];
   ppL = pL;
   ppL[[idx1]] = Sort[(ppL[[idx1]]~TakeAway~pair)~Append~Sort[Flatten[pair]]];
   (*Print[ppL];*)
   res = res~Append~{rate, ppL};
  ];
  res
 ]
MakeCoalEvents[{{}, {}, {{a}, {d}, {b, c}}}];
MakeCoalEvents[{{}, {}, {{a}, {d}, {b, c}}}];
MakeCoalEvents[{{}, {}, {{a}, {d}, {b, c}}}];
```

Printed by Wolfram Mathematica Student Edition

In[30]:=

```
MakeMigEvents[pL_List] := Module[
  {mRate = M},
  (*pl at 0 is anc and migration goes from pop 1 to pop 2 pastward in time*)
  {mRate, {pL⟦1⟧, pL⟦2⟧~TakeAway~{#}, pL⟦3⟧~Append~#}} & /@ pL⟦2⟧
 ]
MakeMigEvents[{ {}, {{a}, {b}}, { {c}, {d}} }];
MakeMigEvents[{ {}, {{a, f}}, { {b, a}, {c}, {d}} }];
MakeMigEvents[ { {{a, b}}, {}, {}}];
```

In[34]:=

```
MakeExodusEvents[pL_List] := Module[
  {exRate = δ, res},
  If[pL⟦1⟧ == {}, res = {exRate, {Flatten[pL, 1], {}, {}}}, res = {}];
  Return[{res}]
 ]
MakeExodusEvents[testState];
```

In[36]:=
```
GFim[ω_, xL_List] := Module[
  {coalList = MakeCoalEvents[xL], migList = MakeMigEvents[xL], exodusList =
    MakeExodusEvents[xL], eventList, branchList = Flatten[xL, 1], totalRates},

  If[xL[[1]] == {},
   eventList = {coalList, migList, exodusList}, eventList = {coalList}];

  eventList = Flatten[eventList, 1];

  totalRates = Total[#[[1]] & /@ eventList];
  (*
  totalRates = Total[Total[#[[1]]&/@#]&/@eventList];
  Print[totalRates];
  totalRates = Total[#[[1]]&/@ Flatten[eventList,1]];*)
  (*Print[totalRates];
  Return[0];*)
  (*totalRates =
    Total[#[[1]]&/@coalList]+  Total[#[[1]]&/@migList] + Total[#[[1]]&/@exodusList];*)
  If[ Length[branchList] == 1, 1,
                        1
   ──────────────────────────────────────── *
   totalRates + Total[ω[#] & /@ branchList]
    ( Total[#[[1]] * GFim[ω, #[[2]]] & /@ eventList])
  ]

  (*Total[(#[[1]]*GFim[ω,#[[2]]])&/@#]&/@eventList*)
  (*
  Total[(#[[1]] * GFim[ω,#[[2]]])&/@coalList]+Total[(#[[1]] * GFim[ω,#[[2]]])&/@migList]+
   Total[(#[[1]] * GFim[ω,#[[2]]])&/@exodusList]*)

 ];
```

The generating function for the IM model and the probability of incongruence

We first obtain the generating function under the IM model for a pair of unphased diploids, one sampled from each population. We expand this so that the GF is a sum of terms, each corresponding to a particular path realized by the coalescent process under the IM model.

We then use substitution to map the branch type labels, ω[{}], onto the unrooted versions. I.e. we cannot distinguish an {a,a,b} branch from a {b} branch type.

In[37]:=
```
gfIM = GFim[ω, {{}, {{a}, {a}}, {{b}, {b}}}] // Expand;
Length[gfIM]
gfIMUnrooted =
  gfIM /. {ω[{a, a, b}] ⧴ ω[{b}], ω[{a, b, b}] ⧴ ω[{a}], ω[{b, b}] ⧴ ω[{a, a}]};
Length[gfIMUnrooted]
```
Out[38]=

95

Out[40]=

95

These are all the branch types observed among all the paths in the IM model, for both the rooted and unrooted genealogies.

In[41]:=
```
GetVars[ω, gfIM]
GetVars[ω, gfIMUnrooted]
```
Out[41]=

{ω[{a}], ω[{b}], ω[{a, a}], ω[{a, b}], ω[{b, b}], ω[{a, a, b}], ω[{a, b, b}]}

Out[42]=

{ω[{a}], ω[{b}], ω[{a, a}], ω[{a, b}]}

For ease, we instead store the terms corresponding to individual paths as elements of a list rather than elements of a series. Each element of the list corresponds to a unique sequence of coalesence, migration, and population split events.

In[43]:=
```
gfList = gfIMUnrooted;
gfList[[0]] = List;
```

Here, we distinguish the branch types present in the unrooted topologies. There are two internal branch types, one of which is incongruent with the species tree. There are two distinguishable external branch types identified by the subpopulation from which the subtending leaf node was sampled.

In[45]:=
```
internalBranchTypes = {ω[{a, a}], ω[{a, b}]};
incongruentBranchTypes = {ω[{a, b}]};
externalBranchTypes = {ω[{a}], ω[{b}]};
```

For each of the paths in the gfList, we extract the branch type variables that are present, check for the presence of an incongruent branch type, then we
take the subset of the paths in the gfList that are indeed incongruent.

In[48]:=
```
PathVars = GetVars[ω, #] & /@ gfList;
incongruentBool = ContainsAny[#, incongruentBranchTypes] & /@ PathVars;
incongruentPaths = Pick[gfList, incongruentBool];
Length[incongruentPaths]
```
Out[51]=

33

We can view individual paths, with terms in the denominator denoting the sequence of states through which the population passes. As an example, consider path number 9 (below)

(2 M + δ + λa + λb + 2 ω[{a}] + 2 ω[{b}]) corresponds to the events that can occur and the branch types that are present for the initial configuration of the sampled lineages under the IM model. This is shared among all paths. We can break this down as follows: two lineages can migrate (2M), the population split could occur (δ), or coalescence could occur in either the A population (λa) or the B population (λb). During this first interval of the coalescent process there are two of each external branch type present: 2 ω[{a}] and 2 ω[{b}].

We can parse the terms in the denominator to determine the sequence of events in this path and the subsequent configurations of the coalescent process. For path number 9, the coalescent history is as follows:

migration from A to B pastward --> (M + δ + 3 λb + 2 ω[{a}] + 2 ω[{b}])
coalescence of an ‘a’ and ‘b’ lineage, generating an incongruent branch type --> (M + δ + λb + ω[{a}] + ω[{b}] + ω[{a, b}])
coalescence of the ‘b’ and ‘ab’ lineage, which results in two ‘a’ branch types when the topology is unrooted --> (M + δ + 2 ω[{a}])
a second migration from A to B pastward --> (δ + λb + 2 ω[{a}])
the split, moving all lineages to the ancestral AB population --> (λab + 2 ω[{a}])
And finally, coalescence of the remaining ‘a’ type lineages.

In[52]:=
```
incongruentPaths[[9]]
```
Out[52]=
```
(4 M^2 δ λab λb^2) /
 ((M + δ + 2 ω[{a}]) (λab + 2 ω[{a}]) (δ + λb + 2 ω[{a}]) (2 M + δ + λa + λb + 2 ω[{a}] + 2 ω[{b}])
   (M + δ + 3 λb + 2 ω[{a}] + 2 ω[{b}]) (M + δ + λb + ω[{a}] + ω[{b}] + ω[{a, b}]))
```

At this point, the discrete event was treated as a competing exponential process, similar to coalescence or migration. We apply the following inverse Laplace transform to each path to obtain the expression as a function of a discrete event occurring at time Td.

In[53]:=
```
incongruentPathsInverted = InverseLaplaceTransform[# / δ, δ, Td] & /@ incongruentPaths;
```

We can obtain the probability of each path by setting the ω branch-length variables to zero, and summing, we obtain the total probability of observing an incongruent genealogy under the IM model. Note that this probability is a function of the size of populations A and B, but not the ancestral population size. I.e. λab does not factor into the expression.

Setting λa and λb to 1 for simplicity, we obtain the probability of incongruence as a function of the migration rate M and split time Td, and we plot this probability as a heatmap as well as for the transect where Td = 1.

Printed by Wolfram Mathematica Student Edition

In[54]:=
```
Pincongo = FullSimplify[Total[incongruentPathsInverted /. {ω[_] → 0}]]
Pincongo /. {λb → 1, λa → 1}
ContourPlot[Pincongo /. {λb → 1, λa → 1} , {Td, 0, 5}, {M, 0, 5},
 PlotLegends → Automatic, PlotPoints → 20, FrameLabel → {"T", "M"}]
Plot[(Pincongo /. {λb → 1, λa → 1, Td → 1}), {M, 0, 10}, AxesLabel → {"M", "Prob"}]
```

Out[54]=

$$\frac{2\, e^{-Td\,(2M+\lambda a+\lambda b)}\left(2\, e^{Td\,(2M+\lambda a+\lambda b)}\, M + \lambda a + \lambda b\right)}{3\,(2M+\lambda a+\lambda b)}$$

Out[55]=

$$\frac{2\, e^{-((2+2M)\,Td)}\left(2 + 2\, e^{(2+2M)\,Td}\, M\right)}{3\,(2+2M)}$$

Out[56]=

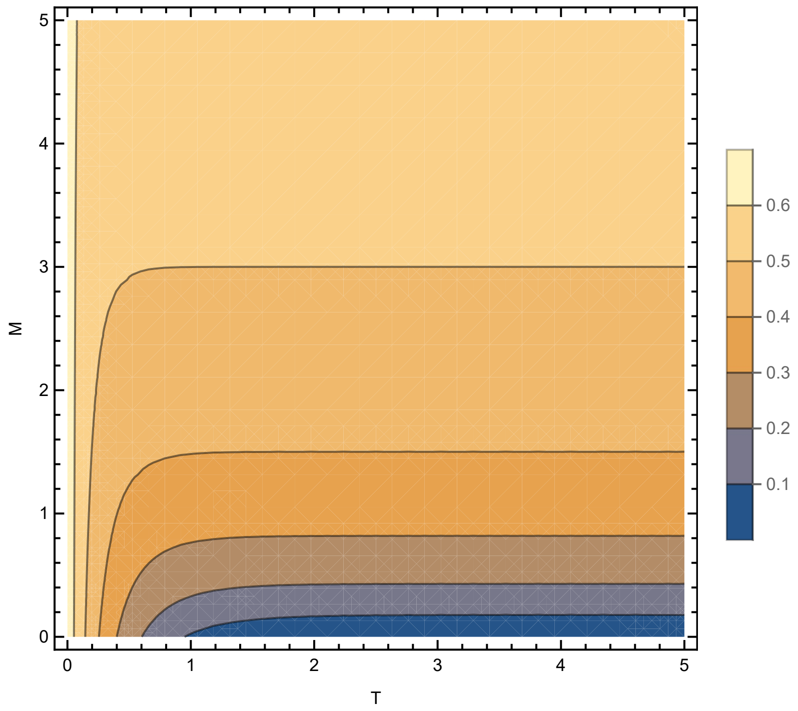

Out[57]=

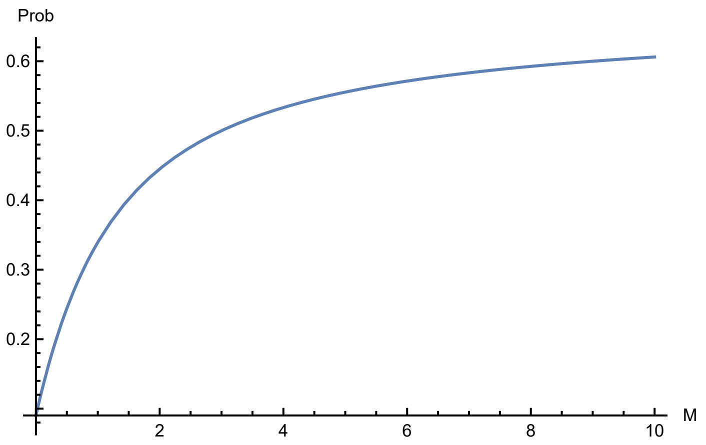

The GF conditioned on incongruent genealogies

To obtain the generating function (here as a list of terms) for incongruent genealogies, we simply retain only the paths that are incongruent, and divide by the total probability of incongruence as derived above.

Setting ω[] terms to zero gives us the probability for each incongruent path

In[58]:=
```
gfIncongruentList = incongruentPathsInverted * 1 / Pincongo;
incongruentPathProbabilities = gfIncongruentList /. ω[_] → 0;
```

To obtain the marginal expected length of external ‘a’ branches (respectively, ‘b’ branches) for each path, we take the derivative with respect to ω[{a}] (respectively, ω[{b}]), multiply by -1, and set all the ω[] variables to 0.

In[60]:=
```
incongruentExpectedTaList = (-1 * D[#, ω[{a}]] & /@ gfIncongruentList) /. ω[_] → 0;
incongruentExpectedTbList = (-1 * D[#, ω[{b}]] & /@ gfIncongruentList) /. ω[_] → 0;
```

Here, we consider the difference in the expected external branch lengths for each path.

For some paths, the expected ‘a’ and ‘b’ branch lengths are equal (i.e. the difference = 0.0). Some paths do have a difference in branch lengths, but they are paired with an equally probable symmetric path, thereby cancelling each other out (e.g. the path pairs (1 & 2) or (5&6). However, there are three paths (indexed as 7,8, and 9 below) that are asymmetrical and produce a measurable difference in the expected external branch lengths.

In[62]:=
```
(incongruentExpectedTaList - incongruentExpectedTbList) /.
 {λb → 2, λa → 1, λab → 1, Td → 1, M → 1.001}
```

Out[62]=
```
{0.011089, -0.011089, 0.00960117, -0.00960117, 0.00128788, -0.00128788,
 0.83656, 0.0545259, 0.294838, 0.0920469, -0.0920469, 0.0108207, 0.0494168,
 -0.0108207, -0.0494168, 0.0063118, -0.0063118, 0.0129278, 0.0863545,
 -0.0129278, -0.0863545, 0.0218327, -0.0218327, 0, 0, 0, 0, 0, 0, 0, 0, 0, 0}
```

We can verify this for the full symbolic expression by checking that the difference in the total expected external branch lengths is 0 if paths 7,8, and 9 are removed.

In[63]:=
```
symmetricPaths = Range[1, 6] ~Join~ Range[10, 33];
Total[incongruentExpectedTaList⟦symmetricPaths⟧] -
 Total[incongruentExpectedTbList⟦symmetricPaths⟧]
```

Out[64]=
```
0
```

Here, assuming parameters {λb -> 1, λa -> 1, Td -> 1, λab -> 1} while allowing M to vary, we plot:

i) the probability of incongruence
ii) the expected total (marginal) length of external branch types ‘a’ and ‘b’ given an incongruent geneal-

ogy

In[65]:=
```
incongruentExpectedTaTotal = Total[incongruentExpectedTaList];
incongruentExpectedTbTotal = Total[incongruentExpectedTbList];
```

In[67]:=
```
Plot[Pincongo /. {λb → 1, λa → 1, Td → 1, λab → 1},
 {M, 0, 5}, PlotRange → {0, 0.66}, AxesLabel → {"M", "Prob"}]
Plot[{incongruentExpectedTaTotal /. {λb → 1, λa → 1, Td → 1, λab → 1},
  incongruentExpectedTbTotal /. {λb → 1, λa → 1, Td → 1, λab → 1}}, {M, 0, 5},
 PlotRange → {{0, 4}, {0, 5}}, AxesLabel → {"M", "Branch lengths"}]
```

Out[67]=

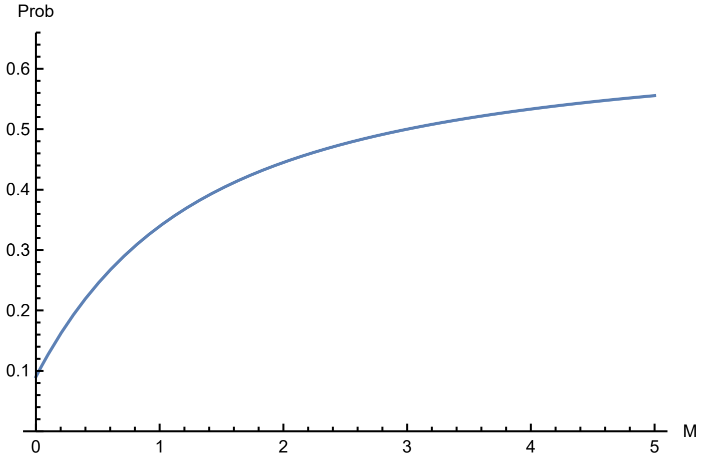

Out[68]=

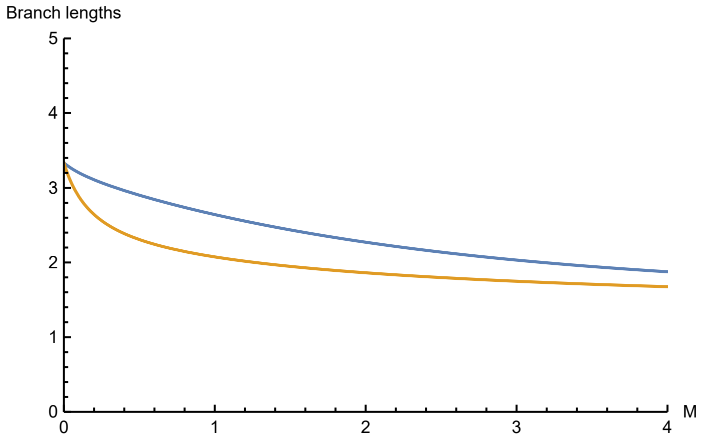

In[69]:=

We can also look at individual incongruent paths. Here, we consider the incongruent asymmetric path number 9 above. We plot

(i) the probability of observing the incongruent path number 9 among any paths
(ii) the probability of observing the asymmetric path number 9 among incongruent paths (i.e. for the GF conditioned on incongruence)
(iii) The expected length of the external branch types conditioned on observing path number 9

In[70]:=
```
pathIndex = 9
pathProbNotConditioned = incongruentPathsInverted[[pathIndex]] /. ω[_] → 0;
pathProbGivenIncongruent = incongruentPathProbabilities[[pathIndex]];
plotset = {PlotStyle → Thick, AxesStyle → Thick,
   FrameStyle → Thick, LabelStyle → {FontSize → 14 , Black, Bold}};

Plot[
 pathProbNotConditioned /. {λb → 1, λa → 1, Td → 1, λab → 1},
 {M, 0, 10},
  PlotRange → {0, 0.055}, PlotStyle → {Purple, Thickness[0.01]},
 Axes → False, Frame → {{True, False}, {True, False}},
 FrameLabel → {{"Path prob not conditioned", None}, {"M", None}},
 FrameTicks → {{{0, 0.02, 0.04}, None}, {{0, 2, 4, 6, 8, 10}, None}}, Evaluate@plotset]

Plot[
 pathProbGivenIncongruent /. {λb → 1, λa → 1, Td → 1, λab → 1},
 {M, 0, 10},
  PlotRange → {0, 0.1}, PlotStyle → {Purple, Thickness[0.01]},
 Axes → False, Frame → {{True, False}, {True, False}},
 FrameLabel → {{"Path Prob given Incongruent", None}, {"M", None}}, FrameTicks →
  {{{0, 0.02, 0.04, 0.06, 0.08}, None}, {{0, 2, 4, 6, 8, 10}, None}}, Evaluate@plotset]

Plot[
 {
  incongruentExpectedTaList[[pathIndex]] / pathProbGivenIncongruent /.
    {λb → 1, λa → 1, Td → 1, λab → 1}, incongruentExpectedTbList[[pathIndex]] /
     pathProbGivenIncongruent /. {λb → 1, λa → 1, Td → 1, λab → 1}
 },
 {M, 0.0001, 10},
 PlotRange → {0, All}, Axes → False, Frame → {{True, False}, {True, False}},
 FrameLabel → {{"Branch lengths τ", None}, {"M", None}},
 FrameTicks → {{{0, 1, 2, 3, 4}, None}, {{0, 2, 4, 6, 8, 10}, None}}, PlotStyle →
  {{Blue, Dashed, Thickness[0.01]}, {Red, Thickness[0.01]}}, Evaluate@plotset]
```

Out[70]=

9

Out[74]=

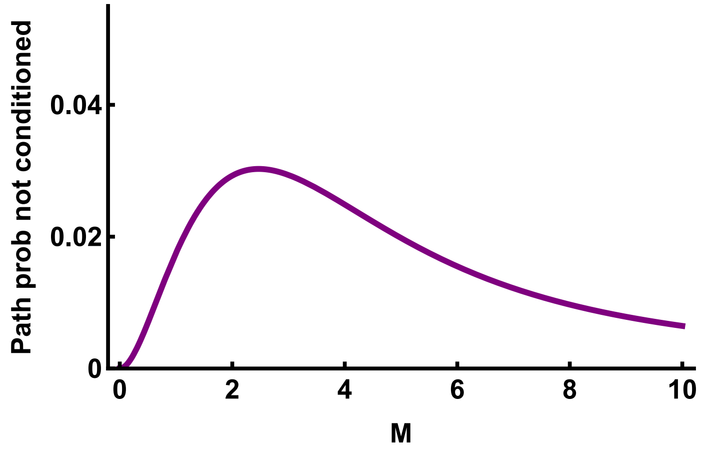

Out[75]=

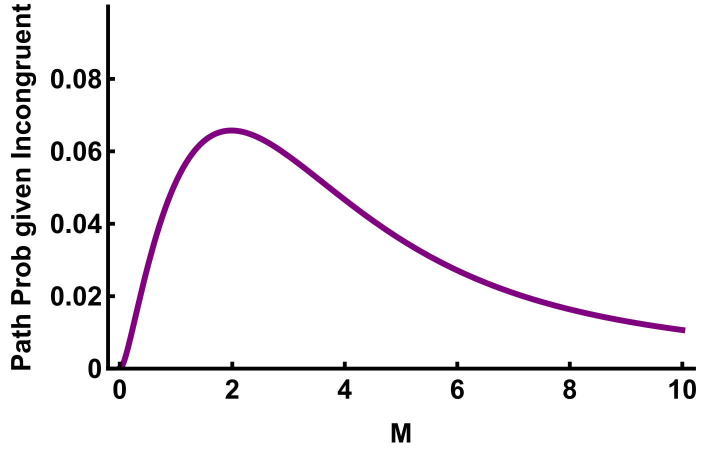

Out[76]=

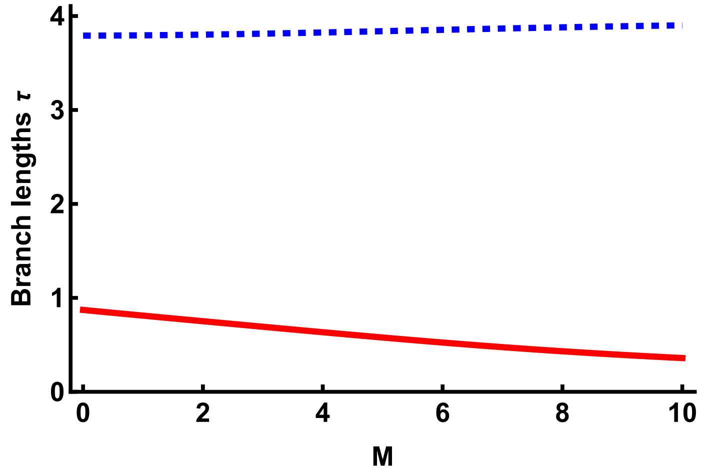

Finally, we can define our test statistic A_i of the asymmetry in incongruent genealogies:

In[77]:=
```
Ai[Td_, M_, λa_, λb_, λab_] = (incongruentExpectedTaTotal - incongruentExpectedTbTotal) /
    (incongruentExpectedTaTotal + incongruentExpectedTbTotal);
```

Here, we provide a contour plot for Ai, setting the λa, λb, and λab parameters to 1

In[78]:=
```
AiAllLambda1 = Ai[Td, M, 1, 1, 1]; (*this makes evaluation for the contour plot faster*)
ContourPlot[AiAllLambda1, {Td, 0, 5}, {M, 0, 2},
 PlotLegends → Automatic, PlotPoints → 20, FrameLabel → {"T", "M"}]
```

Out[79]=

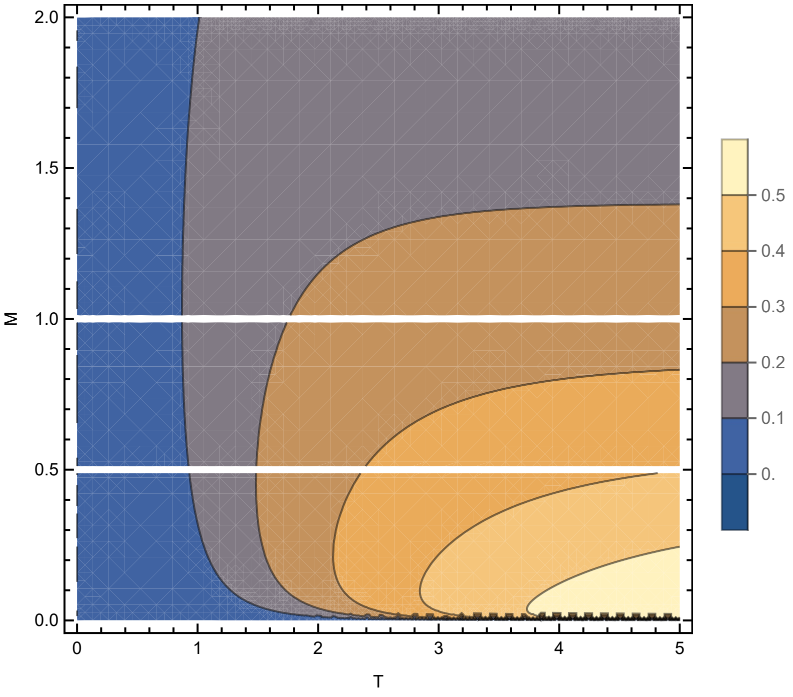

We can try to get a broader view of where A_i = (a - b) / (a + b) is maximised by its relationship with M and T

Very high values of Ai are obtained when T is large and M is very small. However, by calculating the probability of incongruence for such values, we can also see that they will lead to incongruent topologies being very rare. In other words, Ai values close to 1 are unlikely to ever be inferred from genomic data.

In[80]:=
```
Print["Ai               Td          M               Pincongo"]
{#[[1]], #[[2]], Pincongo /. {λa → 1, λb → 1, λab → 1} /. #[[2]]} &/@
 {NMaximize[{AiAllLambda1, 0 < Td < 0.501, 10^(-20) < M < 10},
   {Td, M}, Method → "DifferentialEvolution", MaxIterations → 200]}
{#[[1]], #[[2]], Pincongo /. {λa → 1, λb → 1, λab → 1} /. #[[2]]} &/@
 {NMaximize[{AiAllLambda1, 0 < Td < 1.001, 10^(-20) < M < 10},
   {Td, M}, Method → "DifferentialEvolution", MaxIterations → 200]}
{#[[1]], #[[2]], Pincongo /. {λa → 1, λb → 1, λab → 1} /. #[[2]]} &/@
 {NMaximize[{AiAllLambda1, 0 < Td < 3.001, 10^(-20) < M < 10},
   {Td, M}, Method → "DifferentialEvolution", MaxIterations → 200]}
{#[[1]], #[[2]], Pincongo /. {λa → 1, λb → 1, λab → 1} /. #[[2]]} &/@
 {NMaximize[{AiAllLambda1,  0 < Td < 5.001, 10^(-20) < M < 10},
   {Td, M}, Method → "DifferentialEvolution", MaxIterations → 200]}
{#[[1]], #[[2]], Pincongo /. {λa → 1, λb → 1, λab → 1} /. #[[2]]} &/@
 {NMaximize[{AiAllLambda1,  0 < Td < 10.001, 10^(-20) < M < 10},
   {Td, M}, Method → "DifferentialEvolution", MaxIterations → 200]}
```

```
Ai              Td          M               Pincongo
```

Out[81]=
```
{{0.0439829, {Td → 0.501, M → 2.03834}, 0.457698}}
```

Out[82]=
```
{{0.121088, {Td → 1.001, M → 0.847602}, 0.314769}}
```

Out[83]=
```
{{0.419633, {Td → 3.001, M → 0.0827314}, 0.051867}}
```

Out[84]=
```
{{0.601153, {Td → 5.001, M → 0.00997622}, 0.00661219}}
```

Out[85]=
```
{{0.774572, {Td → 10.001, M → 0.0000607848}, 0.0000405221}}
```

Here we define some functions that will help us evaluate and plot the expressions we have derived above.

In[86]:=
```
evalPincongo[td_, m_, la_, lb_, lab_] :=
  Pincongo /. {ω[_] → 0} /. {Td → td, M → m, λa → la, λb → lb, λab → lab};
evalExpectedBTa[td_, m_, la_, lb_, lab_] :=
  incongruentExpectedTaTotal /. {Td → td, M → m, λa → la, λb → lb, λab → lab};
evalExpectedBTb[td_, m_, la_, lb_, lab_] :=
  incongruentExpectedTbTotal /. {Td → td, M → m, λa → la, λb → lb, λab → lab};
```

Here, we set all λ variables to one, and show for varying values of Td and M:

(i) the probability of incongruence
(ii) expected total external branch lengths for ‘a’ (solid) and ‘b’ (dashed) type branches
(iii) The value of Ai

In[89]:=
```
plotset = {PlotStyle → Thick, AxesStyle → Thick,
   FrameStyle → Thick, LabelStyle → {FontSize → 14 , Black, Bold}};
```

```
In[90]:= Legended[Show[
    Plot[evalPincongo[.501, M, 1, 1, 1], {M, 0, 5}, PlotStyle → {Gray, Thickness[0.01]},
     PlotRange → All, Axes → False, Frame → {{True, False}, {True, False}},
     FrameLabel → {{"Probability of incongruence", None}, {"M", None}}, FrameTicks →
      {{{0, 0.2, 0.4, 0.6}, None}, {{0, 1, 2, 3, 4, 5}, None}}, Evaluate@plotset],
    Plot[evalPincongo[1.001, M, 1, 1, 1], {M, 0, 5},
     PlotStyle → {Purple, Thickness[0.01]}, PlotRange → All],
    Plot[evalPincongo[2.001, M, 1, 1, 1], {M, 0, 5},
     PlotStyle → {Orange, Thickness[0.01]}, PlotRange → All],
    PlotRange → {All, {0, All}}
   ], LineLegend[{Gray, Purple, Orange},
    {"T=0.5", "T=1.0", "T=2.0"}, LabelStyle → {FontSize → 14 , Black, Bold},
    LegendLayout → "Row", LegendMarkerSize → {50, 50, 50} ]]

  Show[
   Plot[evalExpectedBTa[.501, M, 1, 1, 1], {M, 0, 5},
    PlotStyle → {Gray, Thickness[0.01]}, Axes → False, Frame → {{True, False}, {True, False}},
    FrameLabel → {{"Branch lengths", None}, {"M", None}},
    FrameTicks → {{{2, 3, 4, 5}, None}, {{0, 1, 2, 3, 4, 5}, None}},
    Evaluate@plotset, PlotRange → {{0, 5}, {1.5, 5}}],
   Plot[evalExpectedBTa[1.001, M, 1, 1, 1], {M, 0, 5},
    PlotStyle → {Purple, Thickness[0.01]}, PlotRange → {{0, 5}, {1.5, 5}}],
   Plot[evalExpectedBTa[2.001, M, 1, 1, 1], {M, 0, 5},
    PlotStyle → {Orange, Thickness[0.01]}, PlotRange → {{0, 5}, {1.5, 5}}],
   Plot[evalExpectedBTb[.501, M, 1, 1, 1], {M, 0, 5},
    PlotStyle → {Gray, Thickness[0.01], Dashed}, PlotRange → {{0, 5}, {1.5, 5}}],
   Plot[evalExpectedBTb[1.001, M, 1, 1, 1], {M, 0, 5},
    PlotStyle → {Purple, Thickness[0.01], Dashed}, PlotRange → {{0, 5}, {1.5, 5}}],
   Plot[evalExpectedBTb[2.001, M, 1, 1, 1], {M, 0, 5},
    PlotStyle → {Orange, Thickness[0.01], Dashed}, PlotRange → {{0, 5}, {1.5, 5}}]
  ]

  Show[
   Plot[Ai[0.501, M, 1, 1, 1], {M, 0, 5}, PlotStyle → {Gray, Thickness[0.01]},
    PlotRange → All, Axes → False, Frame → {{True, False}, {True, False}},
    FrameLabel → {{"Ai", None}, {"M", None}}, FrameTicks →
     {{{0, 0.1, 0.2, 0.3}, None}, {{0, 1, 2, 3, 4, 5}, None}}, Evaluate@plotset],
   Plot[Ai[1.001, M, 1, 1, 1], {M, 0, 5},
    PlotStyle → {Purple, Thickness[0.01]}, PlotRange → All],
   Plot[Ai[2.001, M, 1, 1, 1], {M, 0, 5},
    PlotStyle → {Orange, Thickness[0.01]}, PlotRange → All],
   PlotRange → {All, {0, All}}
  ]
```

Out[90]=

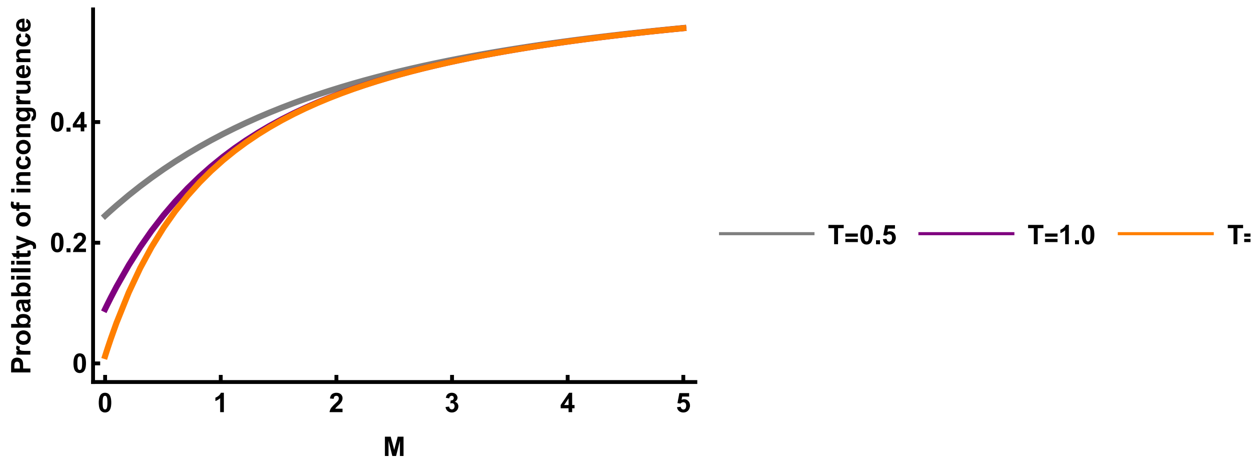

Out[91]=

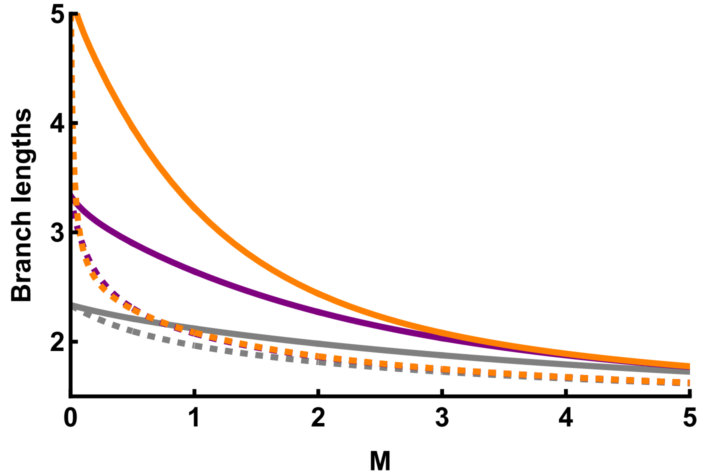

Out[92]=

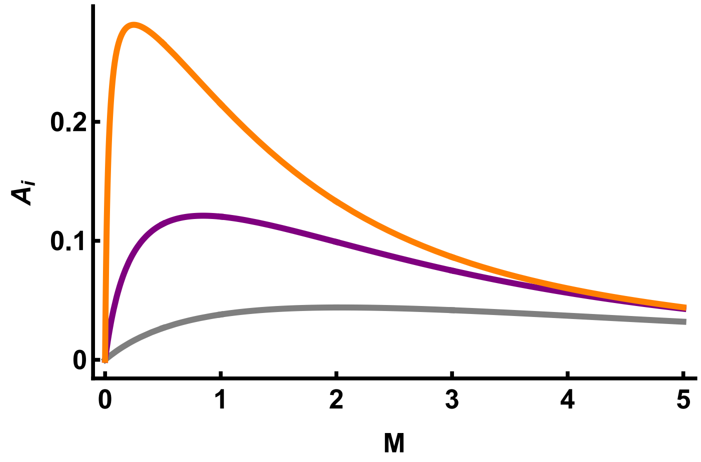

The GF conditioned on observing at least one HetAB type mutation

Here, we obtain the generating function for the process conditioned on the presence of at least one hetAB type mutation.

In[93]:=
```
gfInvertedList = InverseLaplaceTransform[# / δ, δ, Td] & /@ gfList;
```

Define two GFs: (i) the original which gives the probability of coalescence before mutation on all branches, (ii) a GF where the length of the ab branch is marginalised over.

In[94]:=
```
pr0Ha0Hb0fd0HabList = gfInvertedList;
pr0Ha0Hb0fdAnyHabList = # /. ω[{a, b}] → 0 & /@ gfInvertedList;
```

Now subtract GF_ii from GF_i to obtain the probability of coalescence before mutation conditional on at least one hetAB mutation. Dividing by the total probability of observing a hetAB type mutation and retaining the non-zero terms gives the GF conditioned on observing at least one hetAB type mutation.

In[96]:=
```
pr0Ha0Hb0fdGeq1HabList =
  MapThread[#1 - #2 &, {pr0Ha0Hb0fdAnyHabList, pr0Ha0Hb0fd0HabList}];
pr0Ha0Hb0fdGeq1HabList = DeleteCases[pr0Ha0Hb0fdGeq1HabList, 0];
pr0Ha0Hb0fdGeq1HabList = pr0Ha0Hb0fdGeq1HabList /. ω[{a, b}] → θab;
PincongohetAB = Total[pr0Ha0Hb0fdGeq1HabList /. {ω[_] → 0}];

gfGivenHetABList = pr0Ha0Hb0fdGeq1HabList / PincongohetAB;
Total[gfGivenHetABList /. ω[_] → 0 /.
   {λa → 1, λb → 2, λab → 3, Td → 2.2, M → 1.01, θab → .001}] // Simplify
```

Out[101]=
```
1.
```

We obtain the expected branch lengths for this conditioned GF as before:

In[102]:=
```
hetABExpectedTaList = (-1 * D[#, ω[{a}]] & /@ gfGivenHetABList) /. ω[_] → 0;
hetABExpectedTbList = (-1 * D[#, ω[{b}]] & /@ gfGivenHetABList) /. ω[_] → 0;

hetABExpectedTaTotal = Total[hetABExpectedTaList];
hetABExpectedTbTotal = Total[hetABExpectedTbList];
```

We define a few functions to make evaluating these expressions easier

In[106]:=

```
evalPincongohetAB[td_, m_, la_, lb_, lab_, thab_] :=
  PincongohetAB /. {Td → td, M → m, λa → la, λb → lb, λab → lab, θab → thab};
evalhetABExpectedTaTotal[td_, m_, la_, lb_, lab_, thab_] :=
  hetABExpectedTaTotal /. {Td → td, M → m, λa → la, λb → lb, λab → lab, θab → thab};
evalhetABExpectedTbTotal[td_, m_, la_, lb_, lab_, thab_] :=
  hetABExpectedTbTotal /. {Td → td, M → m, λa → la, λb → lb, λab → lab, θab → thab};

evalPincongohetAB [0.51, 1.5, 1.03, 2.33, 1.77, 1.20]
evalhetABExpectedTaTotal[0.51, 1.5, 1.03, 2.33, 1.77, 1.20]
evalhetABExpectedTbTotal[0.51, 1.5, 1.03, 2.33, 1.77, 1.20]
```

Out[109]=

```
0.108922
```

Out[110]=

```
1.30161
```

Out[111]=

```
1.15234
```

Finally, we define our measure of asymmetry conditioned on observing at least one hetAB type mutation Am as follows:

In[112]:=

```
Am[td_, m_, la_, lb_, lab_, thab_] :=
  Module[{A = evalhetABExpectedTaTotal[td, m, la, lb, lab, thab],
    B = evalhetABExpectedTbTotal[td, m, la, lb, lab, thab]},
   Return[ (A - B) / (A + B)]
  ];
```

We also want to know the difference in external branch lengths among all paths, as this is what we would expect if a sequence block is very long and recombination happens at a high rate. We obtain this as above, but this time, we use the full GF for the IM model without any conditioning.

In[113]:=

```
allPathsExptedTa = Total[ -1 * D[#, ω[{a}]] &/@gfInvertedList] /. ω[_] → 0;
evalAllPathsExptedTa[td_, m_, la_, lb_, lab_] :=
  allPathsExptedTa /. {Td → td, M → m, λa → la, λb → lb, λab → lab};

allPathsExptedTb = Total[ -1 * D[#, ω[{b}]] &/@gfInvertedList] /. ω[_] → 0;
evalAllPathsExptedTb[td_, m_, la_, lb_, lab_] :=
  allPathsExptedTb /. {Td → td, M → m, λa → la, λb → lb, λab → lab};

Aall[td_, m_, la_, lb_, lab_] := Module[
   {A = evalAllPathsExptedTa[td, m, la, lb, lab],
    B = evalAllPathsExptedTb[td, m, la, lb, lab]},
   Return[ (A - B) / (A + B)]
  ];
```

Comparison to the simulations and data

In[118]:=

```
Show[
 Plot[Am[1.001, 1.001, 1, 0.5, 1, (block * 2 * 100000 * 10^-8)], {block, 1, 32768 * 2},
  PlotRange → {{0.5, 32768 * 4}, {-0.0125, 0.045}}, ScalingFunctions → {"Log2", None},
  PlotStyle → {LightGray, Thickness[0.02]}, PlotRange → All, Axes → False,
  Frame → {{True, False}, {True, False}}, FrameLabel → {{"Am", None}, {"Block size", None}},
  FrameTicks → {{{-0.01, 0, 0.01, 0.02, 0.03, 0.04}, None},
    {{1, 8, 64, 512, 4096, 65536}, None}}, Evaluate@plotset],
 Plot[Aall[1.001, 1.001, 1, 0.5, 1] + (0 * block), {block, 1, 32768 * 2},
  PlotRange → {{0.5, 32768 * 4}, {-0.0125, 0.045}}, ScalingFunctions → {"Log2", None},
  PlotStyle → {Gray, Thickness[0.02], Dotted}], Graphics[{PointSize[0.0175],
   Point[{{0, 0.009405626769738483}, {1, 0.009364686125991988}, {2, 0.009717606217658794},
     {3, 0.009742735187478662}, {4, 0.01081402914029355}, {5, 0.011770961345514332},
     {6, 0.013868795212550463}, {7, 0.017223201423185255}, {8, 0.023238073753056497},
     {9, 0.02992365865067031}, {10, 0.03590753790710599}, {11, 0.03729532064099975},
     {12, 0.028802239290628195}, {13, 0.012783724106403514}, {14, 0.002153729152671655},
     {15, -0.002119975421769487}, {16, -0.003304655747652981}}],
   Line[{{0, 0.00797025925548094}, {0, 0.010840994283996026}}],
   Line[{{1, 0.007954382479447216}, {1, 0.01077498977253676}}],
   Line[{{2, 0.008269548352500121}, {2, 0.011165664082817467}}],
   Line[{{3, 0.008327916976039556}, {3, 0.011157553398917767}}],
   Line[{{4, 0.00935586008911403}, {4, 0.012272198191473072}}],
   Line[{{5, 0.010335849680406316}, {5, 0.013206073010622349}}],
   Line[{{6, 0.012372580899752322}, {6, 0.015365009525348604}}],
   Line[{{7, 0.015353450200616108}, {7, 0.0190929526457544}}],
   Line[{{8, 0.02115930820197921}, {8, 0.025316839304133785}}],
   Line[{{9, 0.02811763681423012}, {9, 0.03172968048711051}}],
   Line[{{10, 0.03392260227578382}, {10, 0.03789247353842816}}],
   Line[{{11, 0.03514016907058347}, {11, 0.03945047221141604}}],
   Line[{{12, 0.026489585539628806}, {12, 0.031114893041627584}}],
   Line[{{13, 0.009867492315986662}, {13, 0.015699955896820366}}],
   Line[{{14, -0.00078681639433125}, {14, 0.00509427469967456}}],
   Line[{{15, -0.005627953333666082}, {15, 0.001388002490127108}}],
   Line[{{16, -0.007458766018711633}, {16, 0.0008494545234056712}}],
   {Dashed, Line[{{-1, 0}, {17, 0}}]}}]
]
```

Out[118]=

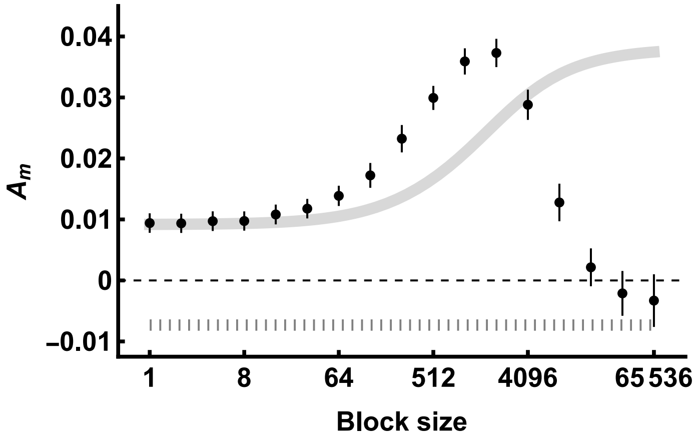

In[119]:=

```
Show[Plot[Am[1.001, 1.001, 1, 1, 1, (block * 2 * 100000 * 10^-8)],
  {block, 1, 32768 * 2}, PlotRange → {{0.5, 32768 * 4}, {-0.025, 0.185}},
  ScalingFunctions → {"Log2", None}, PlotStyle → {LightGray, Thickness[0.02]},
  PlotRange → All, Axes → False, Frame → {{True, False}, {True, False}},
  FrameLabel → {{"Am", None}, {"Block size", None}}, FrameTicks →
   {{{0, 0.04, 0.08, 0.12, 0.16}, None}, {{1, 8, 64, 512, 4096, 65536}, None}},
  Evaluate@plotset], Plot[Aall[1.001, 1.001, 1, 1, 1] + (0 * block),
  {block, 1, 32768 * 2}, PlotRange → {{0.5, 32768 * 4}, {-0.025, 0.185}},
  ScalingFunctions → {"Log2", None}, PlotStyle → {Gray, Thickness[0.02], Dotted}],
 Graphics[{PointSize[0.0175], Point[{{0, 0.033211217314269764}, {1, 0.033504296198764716},
     {2, 0.03387753579407787}, {3, 0.03446992404204243}, {4, 0.03551374891366408},
     {5, 0.037625785327929574}, {6, 0.04214481581313795}, {7, 0.05063660118194881},
     {8, 0.06253862791620005}, {9, 0.08301210401731186}, {10, 0.10815258180427895},
     {11, 0.13298715011931264}, {12, 0.15049714920470175}, {13, 0.1582460163201199},
     {14, 0.15890565931455342}, {15, 0.15955295723975663}, {16, 0.1600362711496164}}],
   Line[{{0, 0.031137381401033747}, {0, 0.03528505322750578}}],
   Line[{{1, 0.03131439329269771}, {1, 0.03569419910483172}}],
   Line[{{2, 0.031835533022364575}, {2, 0.03591953856579117}}],
   Line[{{3, 0.032283731231498386}, {3, 0.036656116852586476}}],
   Line[{{4, 0.03336948768220094}, {4, 0.03765801014512722}}],
   Line[{{5, 0.03534897658336579}, {5, 0.03990259407249336}}],
   Line[{{6, 0.040014790236298094}, {6, 0.0442748413899778}}],
   Line[{{7, 0.04832531320047061}, {7, 0.052947889163427006}}],
   Line[{{8, 0.060109731540578394}, {8, 0.0649675242918217}}],
   Line[{{9, 0.08064194181930809}, {9, 0.08538226621531564}}],
   Line[{{10, 0.10554821444166909}, {10, 0.1107569491668888}}],
   Line[{{11, 0.12977187900800338}, {11, 0.1362024212306219}}],
   Line[{{12, 0.14725890340143447}, {12, 0.15373539500796904}}],
   Line[{{13, 0.15527537378024633}, {13, 0.16121665885999345}}],
   Line[{{14, 0.15550741778955085}, {14, 0.16230390083955598}}],
   Line[{{15, 0.1560109075718947}, {15, 0.16309500690761855}}],
   Line[{{16, 0.15625881527928753}, {16, 0.1638137270199453}}],
   {Dashed, Line[{{-1, 0}, {17, 0}}]}}]]
```

Out[119]=

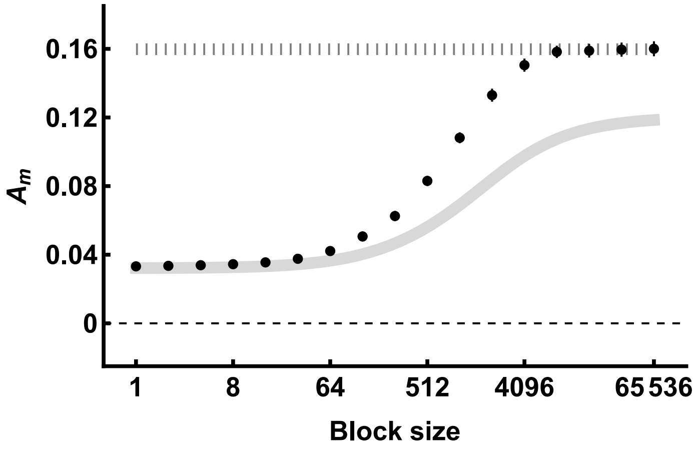

In[120]:=

```
Show[Plot[Am[1.001, 0.001, 0.5, 1, 1, (block * 2 * 100000 * 10^-8)],
  {block, 1, 32768 * 2}, PlotRange → {{0.5, 32768 * 4}, {-0.025, 0.185}},
  ScalingFunctions → {"Log2", None}, PlotStyle → {LightGray, Thickness[0.02]},
  PlotRange → All, Axes → False, Frame → {{True, False}, {True, False}},
  FrameLabel → {{"Am", None}, {"Block size", None}}, FrameTicks →
   {{{0, 0.04, 0.08, 0.12, 0.16}, None}, {{1, 8, 64, 512, 4096, 65536}, None}},
  Evaluate@plotset], Plot[All[1.001, 0.001, 0.5, 1, 1] + (0 * block),
  {block, 1, 32768 * 2}, PlotRange → {{0.5, 32768 * 4}, {-0.025, 0.185}},
  ScalingFunctions → {"Log2", None},
  PlotStyle → {Gray, Thickness[0.02], Dotted}], Graphics[
  {PointSize[0.0175], Point[{{0, -0.0008372321013400357}, {1, -0.0006984806776005199},
     {2, -0.0007935415452194512}, {3, -0.0003092124472274558}, {4, 0.0001550736799263849},
     {5, 0.0010116323475595208}, {6, 0.002520353341291474}, {7, 0.006384814675107434},
     {8, 0.01322952242610933}, {9, 0.026411949209231268}, {10, 0.04746516950367775},
     {11, 0.07606169460313927}, {12, 0.1079939423503988}, {13, 0.1355745187529661},
     {14, 0.15676006318957408}, {15, 0.1671604679684583}, {16, 0.17141690521310068}}],
   Line[{{0, -0.002229953942102494}, {0, 0.0005554897394224226}}],
   Line[{{1, -0.002079040876877616}, {1, 0.0006820795216765761}}],
   Line[{{2, -0.002063959305202264}, {2, 0.0004768762147633614}}],
   Line[{{3, -0.0017906599258153857}, {3, 0.0011722350313604743}}],
   Line[{{4, -0.0013407301138141717}, {4, 0.0016508774736669414}}],
   Line[{{5, -0.0008852297743006503}, {5, 0.0029084944694196917}}],
   Line[{{6, 0.0006661673310707633}, {6, 0.004374539351512185}}],
   Line[{{7, 0.004170132750663594}, {7, 0.008599496599551275}}],
   Line[{{8, 0.010071169388087407}, {8, 0.016387875464131254}}],
   Line[{{9, 0.023842945015164166}, {9, 0.02898095340329837}}],
   Line[{{10, 0.04451660361433006}, {10, 0.05041373539302543}}],
   Line[{{11, 0.073060349606245}, {11, 0.07906303960003355}}],
   Line[{{12, 0.10500441280682628}, {12, 0.11098347189397133}}],
   Line[{{13, 0.13267080099081277}, {13, 0.13847823651511945}}],
   Line[{{14, 0.15375321969430714}, {14, 0.15976690668484103}}],
   Line[{{15, 0.16426360765067005}, {15, 0.17005732828624653}}],
   Line[{{16, 0.168491554696573}, {16, 0.17434225572962836}}],
   {Dashed, Line[{{-1, 0}, {17, 0}}]}}]]
```

Out[120]=

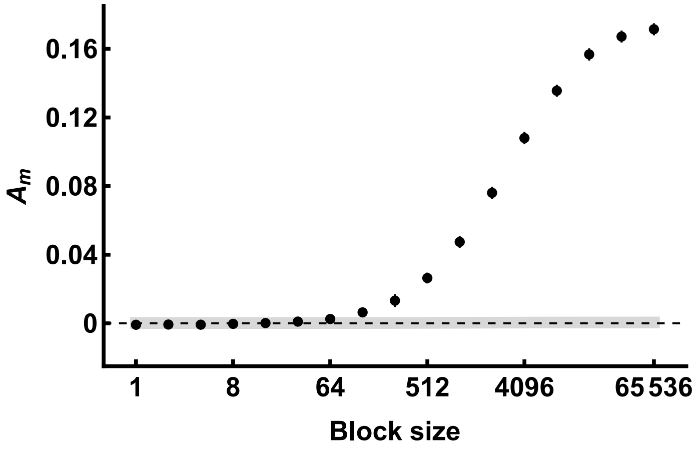

In[121]:=

```
Show[Plot[10 * M, {M, 10, 100}, PlotRange → {{-0.1, 1.6}, {-0.052, 0.052}},
  PlotStyle → {LightGray, Thickness[0.02]}, PlotRange → All, Axes → False,
  Frame → {{True, False}, {True, False}}, FrameLabel → {{"Am", None}, {"MB", None}},
  FrameTicks → {{{-0.04, -0.02, 0, 0.02, 0.04}, None},
    {{0, 0.25, 0.5, 0.75, 1, 1.25, 1.5}, None}},
  ImageSize → {600, 200} , AspectRatio → Full, Evaluate@plotset],
 Graphics[{PointSize[0.014], Orange, Point[{{0.06, 0.038522473886},
     {0.31, 0.022823497940}, {0.56, 0.012567614576}, {0.81, 0.006474568825},
     {1.06, 0.004506934267}, {1.31, -0.000266792021}, {1.56, -0.001408802686}}],
   Line[{{0.06, 0.042558730411}, {0.06, 0.034486217360}}],
   Line[{{0.31, 0.026576013163}, {0.31, 0.019070982717}}],
   Line[{{0.56, 0.015999719787}, {0.56, 0.009135509364}}],
   Line[{{0.81, 0.009998327123}, {0.81, 0.002950810527}}],
   Line[{{1.06, 0.007763949797}, {1.06, 0.001249918736}}],
   Line[{{1.31, 0.003130314011}, {1.31, -0.003663898053}}],
   Line[{{1.56, 0.002302039275}, {1.56, -0.005119644647}}],
   {Dashed, Line[{{-1, 0}, {17, 0}}]}}],

 Plot[10 * M, {M, 10, 100}, PlotRange → {{-0.25, 1.75}, {-0.05, 0.05}},
  PlotStyle → {LightGray, Thickness[0.02]}, PlotRange → All, Axes → False,
  Frame → {{True, False}, {True, False}}, FrameLabel → {{"Am", None}, {"MB", None}},
  FrameTicks → {{{-0.04, -0.02, 0, 0.02, 0.04}, None},
    {{0, 0.25, 0.5, 0.75, 1, 1.25, 1.5}, None}},
  ImageSize → {600, 200} , AspectRatio → Full, Evaluate@plotset],
 Graphics[{PointSize[0.014], Blue, Point[{{0.02, 0.042604746775},
     {0.27, 0.020421212014}, {0.52, 0.010555442624}, {0.77, 0.004542323157},
     {1.02, 0.002824841192}, {1.27, -0.003289867249}, {1.52, -0.003664266800}}],
   Line[{{0.02, 0.038646454495}, {0.02, 0.046563039055}}],
   Line[{{0.27, 0.016633312891}, {0.27, 0.024209111138}}],
   Line[{{0.52, 0.006915259219}, {0.52, 0.014195626028}}],
   Line[{{0.77, 0.001230140474}, {0.77, 0.007854505840}}],
   Line[{{1.02, -0.000654459111}, {1.02, 0.006304141495}}],
   Line[{{1.27, -0.006647203705}, {1.27, 0.000067469207}}],
```

```
   Line[{{1.52, -0.006702319506}, {1.52, -0.000626214094}}],
   {Dashed, Line[{{-1, 0}, {17, 0}}]}}],

 Plot[10 * M, {M, 10, 100}, PlotRange → {{-0.25, 1.75}, {-0.05, 0.05}},
  PlotStyle → {LightGray, Thickness[0.02]}, PlotRange → All, Axes → False,
  Frame → {{True, False}, {True, False}}, FrameLabel → {{"Am", None}, {"MB", None}},
  FrameTicks → {{{-0.04, -0.02, 0, 0.02, 0.04}, None},
    {{0, 0.25, 0.5, 0.75, 1, 1.25, 1.5}, None}},
  ImageSize → {600, 200} , AspectRatio → Full, Evaluate@plotset],
 Graphics[{PointSize[0.014], Point[{{-0.02, 0.042619090875},
     {0.23, 0.015103676429}, {0.48, -0.000539166999}, {0.73, -0.007326869877},
     {0.98, -0.011818402204}, {1.23, -0.015067860357}, {1.48, -0.015365168994}}],
   Line[{{-0.02, 0.038446044963}, {-0.02, 0.046792136787}}],
   Line[{{0.23, 0.010781246883}, {0.23, 0.019426105976}}],
   Line[{{0.48, -0.004246661319}, {0.48, 0.003168327321}}],
   Line[{{0.73, -0.010404149915}, {0.73, -0.004249589838}}],
   Line[{{0.98, -0.015113466291}, {0.98, -0.008523338117}}],
   Line[{{1.23, -0.018454155955}, {1.23, -0.011681564759}}],
   Line[{{1.48, -0.018855887476}, {1.48, -0.011874450512}}],
   {Dashed, Line[{{-1, 0}, {17, 0}}]}}],

 Plot[10 * M, {M, 10, 100}, PlotRange → {{-0.25, 1.75}, {-0.05, 0.05}},
  PlotStyle → {LightGray, Thickness[0.02]}, PlotRange → All, Axes → False,
  Frame → {{True, False}, {True, False}}, FrameLabel → {{"Am", None}, {"MB", None}},
  FrameTicks → {{{-0.04, -0.02, 0, 0.02, 0.04}, None},
    {{0, 0.25, 0.5, 0.75, 1, 1.25, 1.5}, None}},
  ImageSize → {600, 200} , AspectRatio → Full, Evaluate@plotset],
 Graphics[{PointSize[0.014], Pink, Point[{{-0.06, -0.001898626970},
     {0.19, -0.032569545532}, {0.44, -0.042880124935}, {0.69, -0.044758772025},
     {0.94, -0.043794336000}, {1.19, -0.040087162813}, {1.44, -0.039109956878}}],
   Line[{{-0.06, -0.006819718841}, {-0.06, 0.003022464901}}],
   Line[{{0.19, -0.037922350449}, {0.19, -0.027216740616}}],
   Line[{{0.44, -0.047289187545}, {0.44, -0.038471062325}}],
   Line[{{0.69, -0.049185438255}, {0.69, -0.040332105796}}],
   Line[{{0.94, -0.047598140627}, {0.94, -0.039990531373}}],
   Line[{{1.19, -0.044170537462}, {1.19, -0.036003788164}}],
   Line[{{1.44, -0.042693430081}, {1.44, -0.035526483675}}],
   {Dashed, Line[{{-1, 0}, {17, 0}}]}}]]
```

Out[121]=

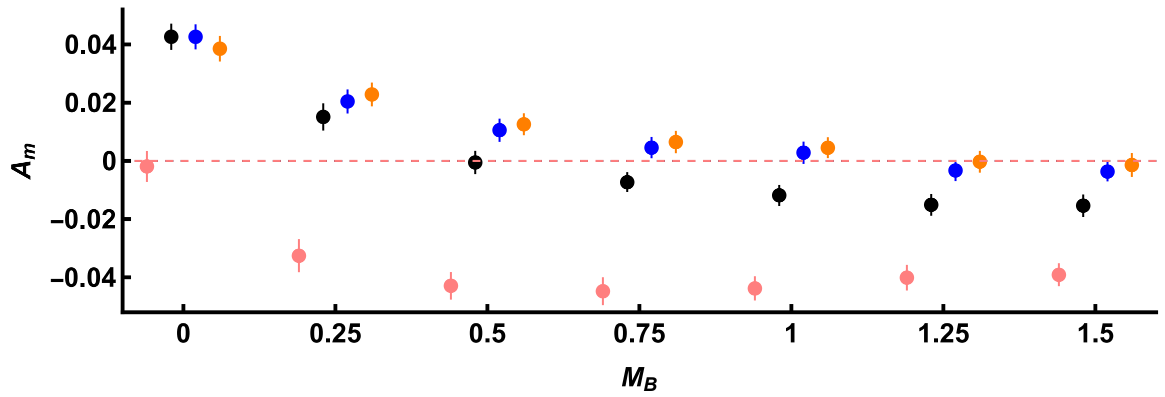

In[122]:=

```
(*Heliconius*)
Show[Plot[10 * M, {M, 10, 100}, PlotRange → {{0.5, 131072}, {-0.065, 0.1}},
  PlotStyle → {LightGray, Thickness[0.02]}, PlotRange → All, Axes → False,
  Frame → {{True, False}, {True, False}}, FrameLabel → {{"Am", None}, {"Block size", None}},
  FrameTicks → {{{-0.04, 0, 0.04, 0.08}, None}, {{1, 8, 64, 512, 4096, 65536}, None}},
  ScalingFunctions → {"Log2", None}, Evaluate@plotset],  Graphics[
  {PointSize[0.0175], Black, Point[{{N[Log2[5]], 0.020642}, {N[Log2[9]], 0.025585},
     {N[Log2[17]], 0.033988}, {N[Log2[33]], 0.042552}, {6, 0.053680}, {7, 0.062154},
     {8, 0.064760}, {9, 0.058785}, {10, 0.042406}, {11, 0.017708}, {12, -0.008113},
     {13, -0.027187}, {14, -0.038115}, {15, -0.042669}, {16, -0.043712}}],
   Line[{{N[Log2[5]], 0.013583}, {N[Log2[5]], 0.027700}}],
   Line[{{N[Log2[9]], 0.019039}, {N[Log2[9]], 0.032132}}],
   Line[{{N[Log2[17]], 0.026966}, {N[Log2[17]], 0.041011}}],
   Line[{{N[Log2[33]], 0.036248}, {N[Log2[33]], 0.048856}}],
   Line[{{6, 0.047267}, {6, 0.060093}}], Line[{{7, 0.055048}, {7, 0.069261}}],
   Line[{{8, 0.056772}, {8, 0.072748}}], Line[{{9, 0.049698}, {9, 0.067873}}],
   Line[{{10, 0.032095}, {10, 0.052717}}], Line[{{11, 0.005669}, {11, 0.029747}}],
   Line[{{12, -0.022159}, {12, 0.005933}}], Line[{{13, -0.043023}, {13, -0.011352}}],
   Line[{{14, -0.054732}, {14, -0.021499}}], Line[{{15, -0.059549}, {15, -0.025789}}],
   Line[{{16, -0.060650}, {16, -0.026773}}], {Dashed, Line[{{-1, 0}, {17, 0}}]}}],

 Plot[10 * M, {M, 10, 100}, PlotRange → {{0.5, 131072}, {-0.065, 0.1}},
  PlotStyle → {LightGray, Thickness[0.02]}, PlotRange → All, Axes → False,
  Frame → {{True, False}, {True, False}}, FrameLabel → {{"Am", None}, {"Block size", None}},
  FrameTicks → {{{-0.04, 0, 0.04, 0.08}, None}, {{1, 8, 64, 512, 4096, 65536}, None}},
  ScalingFunctions → {"Log2", None}, Evaluate@plotset],  Graphics[
  {PointSize[0.0175], Gray, Point[{{0, 0.036804603931241464}, {1, 0.0398171438280715},
     {2, 0.04383603126151459}, {3, 0.050278418914730026}, {4, 0.05925170582857248},
     {5, 0.07233514886714391}, {6, 0.08674758809390354}, {7, 0.0962976655354229},
     {8, 0.0936350204010276}, {9, 0.0748919950328187}, {10, 0.0495187209109811},
     {11, 0.02942959169629271}, {12, 0.020835403460266834}, {13, 0.018519202717663847},
     {14, 0.017286744261448588}, {15, 0.01656075426707176}, {16, 0.016125290326433273}}],
   Line[{{0, 0.03471518966199503}, {0, 0.0388940182004879}}],
   Line[{{1, 0.03774794068271003}, {1, 0.04188634697343297}}],
```

```
    Line[{{2, 0.041634814548063455}, {2, 0.04603724797496573}}],
    Line[{{3, 0.04768822379582485}, {3, 0.0528686140336352}}],
    Line[{{4, 0.056389313443698776}, {4, 0.06211409821344619}}],
    Line[{{5, 0.06991834262005679}, {5, 0.07475195511423104}}],
    Line[{{6, 0.08450758375232734}, {6, 0.08898759243547974}}],
    Line[{{7, 0.09429732277278037}, {7, 0.09829800829806544}}],
    Line[{{8, 0.09117433394109997}, {8, 0.09609570686095523}}],
    Line[{{9, 0.07221857185836594}, {9, 0.07756541820727145}}],
    Line[{{10, 0.04670242817766195}, {10, 0.052335013644300245}}],
    Line[{{11, 0.026361693368166034}, {11, 0.03249749002441938}}],
    Line[{{12, 0.01717212760755346}, {12, 0.024498679312980207}}],
    Line[{{13, 0.014672063562308284}, {13, 0.02236634187301941}}],
    Line[{{14, 0.013435203808553082}, {14, 0.021138284714344095}}],
    Line[{{15, 0.012327533551090633}, {15, 0.02079397498305289}}],
    Line[{{16, 0.011899915589410868}, {16, 0.02035066506345568}}],
    {Dashed, Line[{{-1, 0}, {17, 0}}]}}]]
```

Out[122]=

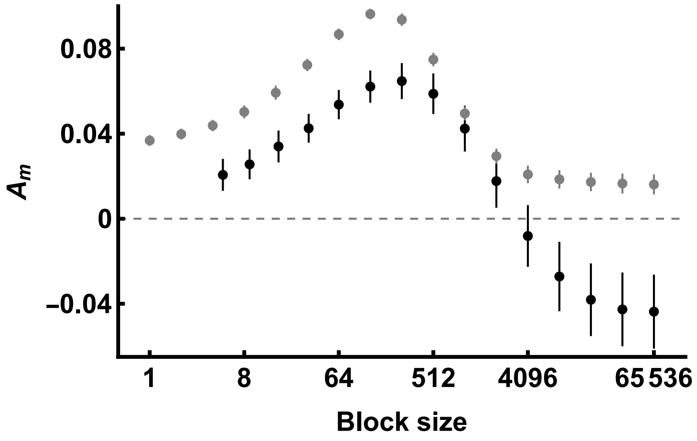

In[128]:=

```
(*Deer*)
Show[Plot[10 * M, {M, 10, 100}, PlotRange → {{0.5, 131072}, {-0.02, 0.38}},
  PlotStyle → {LightGray, Thickness[0.02]}, PlotRange → All, Axes → False,
  Frame → {{True, False}, {True, False}}, FrameLabel → {{"Am", None}, {"Block size", None}},
  FrameTicks → {{{0, 0.12, 0.24, 0.36}, None}, {{1, 8, 64, 512, 4096, 65536}, None}},
  ScalingFunctions → {"Log2", None}, Evaluate@plotset], Graphics[
  {PointSize[0.0175], Black, Point[{{N[Log2[5]], 0.031745}, {N[Log2[9]], 0.032780},
     {N[Log2[17]], 0.041434}, {N[Log2[33]], 0.051257}, {6, 0.066818}, {7, 0.088169},
     {8, 0.121102}, {9, 0.164282}, {10, 0.214786}, {11, 0.265836}, {12, 0.310081},
     {13, 0.340907}, {14, 0.356432}, {15, 0.360029}, {16, 0.359490}}],
   Line[{{N[Log2[5]], 0.022524}, {N[Log2[5]], 0.040965}}],
   Line[{{N[Log2[9]], 0.023484}, {N[Log2[9]], 0.042075}}],
   Line[{{N[Log2[17]], 0.032414}, {N[Log2[17]], 0.050453}}],
   Line[{{N[Log2[33]], 0.043276}, {N[Log2[33]], 0.059237}}],
   Line[{{6, 0.059067}, {6, 0.074569}}], Line[{{7, 0.080322}, {7, 0.096015}}],
   Line[{{8, 0.113062}, {8, 0.129142}}], Line[{{9, 0.155959}, {9, 0.172604}}],
   Line[{{10, 0.206538}, {10, 0.223033}}], Line[{{11, 0.257912}, {11, 0.273761}}],
```

```
   Line[{{12, 0.302717}, {12, 0.317445}}], Line[{{13, 0.334026}, {13, 0.347788}}],
   Line[{{14, 0.349599}, {14, 0.363266}}], Line[{{15, 0.353085}, {15, 0.366973}}],
   Line[{{16, 0.352466}, {16, 0.366514}}], {Dashed, Line[{{-1, 0}, {17, 0}}]}}],

Plot[10 * M, {M, 10, 100}, PlotRange → {{0.5, 131072}, {-0.02, 0.38}},
  PlotStyle → {LightGray, Thickness[0.02]}, PlotRange → All, Axes → False,
  Frame → {{True, False}, {True, False}}, FrameLabel → {{"Am", None}, {"Block size", None}},
  FrameTicks → {{{0, 0.12, 0.24, 0.36}, None}, {{1, 8, 64, 512, 4096, 65536}, None}},
  ScalingFunctions → {"Log2", None}, Evaluate@plotset], Graphics[
  {PointSize[0.0175], Gray, Point[{{0, 0.00410292135906297З}, {1, 0.004714972420580432},
     {2, 0.005221555737208986}, {3, 0.005804516738621062}, {4, 0.005758291409833588}5},
     {5, 0.00673530620596593}, {6, 0.01033620054699807}, {7, 0.017456895000386972},
     {8, 0.0263619271725918}, {9, 0.05310217148034448}, {10, 0.09462734329076124},
     {11, 0.15530805238948533}, {12, 0.2234031181883359}, {13, 0.28699795751461554},
     {14, 0.336897596687793}, {15, 0.36199705882500866}, {16, 0.368786243086038}}],
   Line[{{0, -0.002507806222527876}, {0, 0.010713648940653821}}],
   Line[{{1, -0.001620076431767128}, {1, 0.011050021272927993}}],
   Line[{{2, -0.001150911124592321}, {2, 0.011594022599010292}}],
   Line[{{3, -0.000644898539729901}, {3, 0.012253932016972025}}],
   Line[{{4, -0.0011773246796197914}, {4, 0.012693907499286968}}],
   Line[{{5, -0.0007194225055153674}, {5, 0.014190034917447227}}],
   Line[{{6, 0.0023803480288232017}, {6, 0.01829205306517294}}],
   Line[{{7, 0.008912996090122522}, {7, 0.026000793910651424}}],
   Line[{{8, 0.018606139488304668}, {8, 0.03411771485687893}}],
   Line[{{9, 0.04468931698631741}, {9, 0.06151502597437156}}],
   Line[{{10, 0.08408003181845423}, {10, 0.10517465476306824}}],
   Line[{{11, 0.14442439985548688}, {11, 0.16619170492348379}}],
   Line[{{12, 0.2119356372502968}, {12, 0.23487059912637498}}],
   Line[{{13, 0.27583489689781565}, {13, 0.29816101813141543}}],
   Line[{{14, 0.32573308146741464}, {14, 0.3480621119081714}}],
   Line[{{15, 0.3509081039273523}, {15, 0.37308601372266503}}],
   Line[{{16, 0.3571864286901051}, {16, 0.38038605748197096}}],
   {Dashed, Line[{{-1, 0}, {17, 0}}]}}],

Plot[10 * M, {M, 10, 100}, PlotRange → {{0.5, 131072}, {-0.02, 0.38}},
  PlotStyle → {LightGray, Thickness[0.02]}, PlotRange → All, Axes → False,
  Frame → {{True, False}, {True, False}}, FrameLabel → {{"Am", None}, {"Block size", None}},
  FrameTicks → {{{0, 0.12, 0.24, 0.36}, None}, {{1, 8, 64, 512, 4096, 65536}, None}},
  ScalingFunctions → {"Log2", None}, Evaluate@plotset], Graphics[
  {PointSize[0.0175], Orange, Point[{{0, 0.0194349440010378}, {1, 0.019588591281046466},
     {2, 0.020337470011752984}, {3, 0.02088536134541027}, {4, 0.021725522888452666},
     {5, 0.024191820076171683}, {6, 0.029116366599859094}, {7, 0.03618569033806793},
     {8, 0.04550590505126488}, {9, 0.06883379332725152}, {10, 0.09641376771444339},
     {11, 0.135906393028501}, {12, 0.17976402398980326}, {13, 0.2137318397385111},
     {14, 0.24830704938866086}, {15, 0.27256004657626254}, {16, 0.28481990556016706}}],
   Line[{{0, 0.01590866668474297}, {0, 0.022961221317332633}}],
   Line[{{1, 0.015862407285856004}, {1, 0.02331477527623693}}],
```

```
    Line[{{2, 0.016307500317091342}, {2, 0.024367439706414626}}],
    Line[{{3, 0.01674689304467263}, {3, 0.025023829646147914}}],
    Line[{{4, 0.01734794852578651}, {4, 0.026103097251118822}}],
    Line[{{5, 0.019617135237894726}, {5, 0.02876650491444864}}],
    Line[{{6, 0.024268023705353817}, {6, 0.03396470949436437}}],
    Line[{{7, 0.0297283172947103}, {7, 0.04264306338142556}}],
    Line[{{8, 0.03798937261850527}, {8, 0.05302243748402449}}],
    Line[{{9, 0.06055689747347317}, {9, 0.07711068918102987}}],
    Line[{{10, 0.08666270736836519}, {10, 0.10616482806052159}}],
    Line[{{11, 0.12752178272549006}, {11, 0.14429100333151193}}],
    Line[{{12, 0.17176827665674504}, {12, 0.18775977132286148}}],
    Line[{{13, 0.20396772403579985}, {13, 0.22349595544122233}}],
    Line[{{14, 0.24008305466534477}, {14, 0.2565310441119769}}],
    Line[{{15, 0.2631100103292695}, {15, 0.28201008282325557}}],
    Line[{{16, 0.2766318405255902}, {16, 0.2930079705947439}}],
  {Dashed, Line[{{-1, 0}, {17, 0}}]}}]]
```

Out[128]=

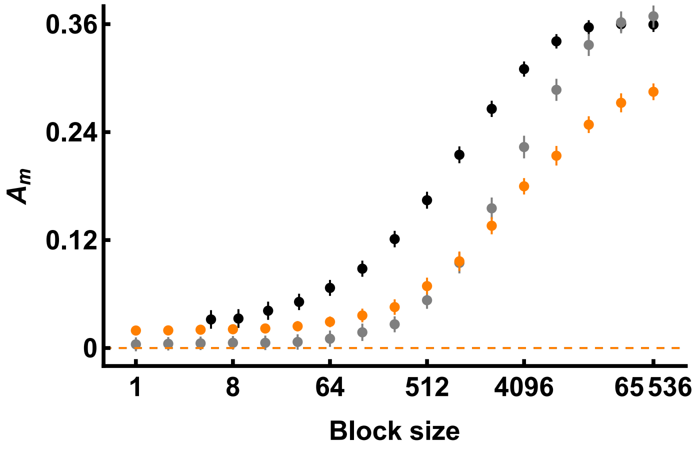

Supplement: iyae157_Supplementary_Data [file iyae157_supplementary_data.zip › File_S1_GENETICS-2024-307366.pdf]
