# Supplementary material for: Genealogical asymmetry under the isolation with migration model and a two-taxon test for gene flow
Source: Genetics. 2024 Sep 30;228(4):iyae157. doi: 10.1093/genetics/iyae157 (PMC11631468; doi:10.1093/genetics/iyae157)
Supplement: iyae157_Supplementary_Data [file iyae157_supplementary_data.zip › Supplemental_Figures_GENETICS-2024-307366.pdf]

Genealogical asymmetry under the IM model and a two-taxon test for gene flow: Supplementary Figures

Alexander Mackintosh*[,1,2] and Derek Setter[2]

[1]Department of Ecology and Genetics, Evolutionary Biology Centre, Uppsala University, Uppsala, Sweden

[2]Institute of Ecology and Evolution, University of Edinburgh, Edinburgh, EH9 3FL, UK

∗ Corresponding author: alexander.mackintosh@ebc.uu.se

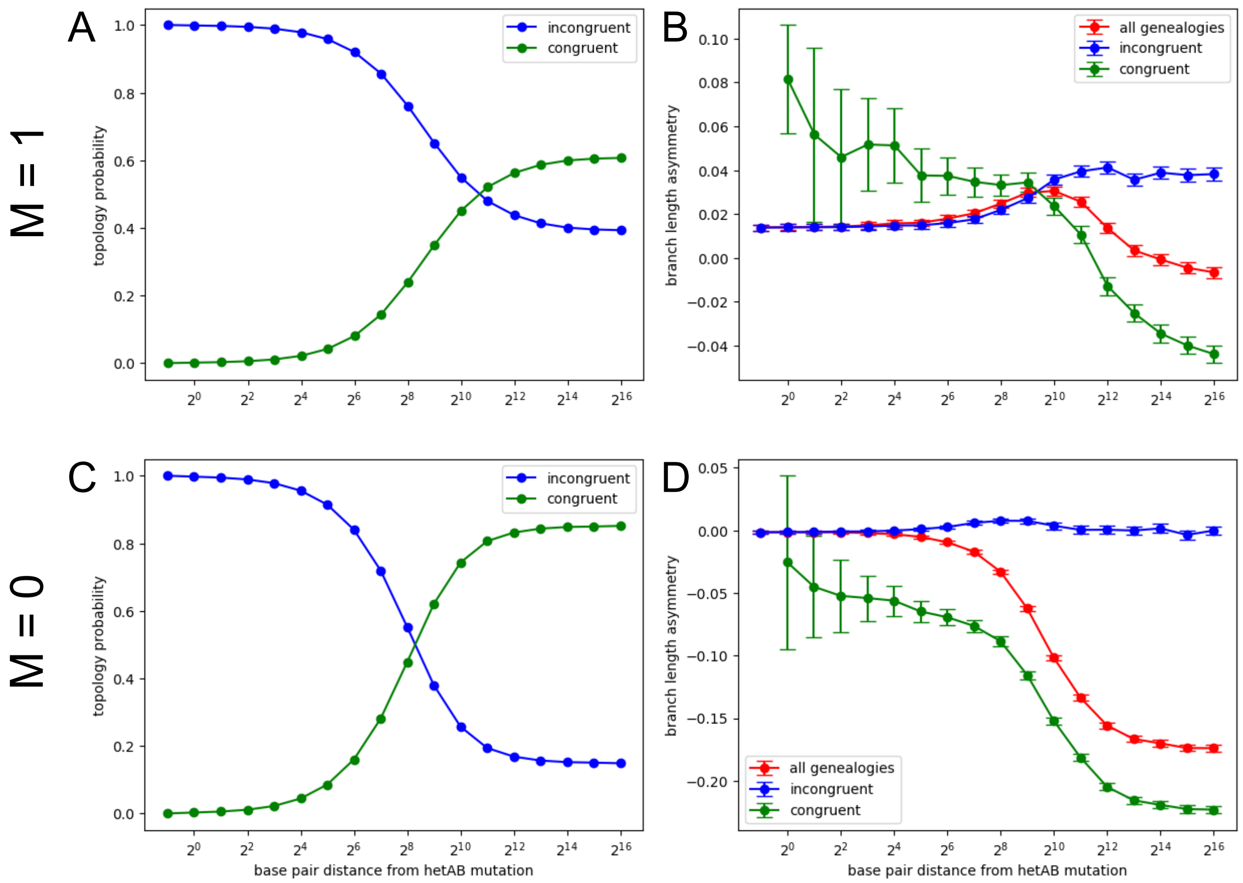

Figure S1: The effect of recombination on (**A** and **C**) the probability of observing incongruent (blue) or congruent (green) topologies and (**B** and **D**) the branch-length asymmetry among incongruent (blue), congruent (green), or all (red) topologies as a function of base-pair distance from an observed het_{AB} type mutation at position 0 (represented here by the left-most point on the log-2 scaled x-axis). Panels **A** and **B** correspond to unidirectional migration from B to A forwards in time at rate $M = 1$; **C** and **D**, to $M = 0$. The remaining parameters match those of Figure 4 panel C: unequal population sizes $N_A = N_{AB} = 100,000$ and $N_B = 200,000$, divergence time $T = 1.0$, per-base mutation rate $\mu = 10^{-8}$, and recombination rate $r = 10^{-8}$ per-base per-generation. Error bars correspond to the 95% CIs of each estimate. Note that no congruent topologies can be present at position 0 given that a het_{AB} mutation is observed at that site.

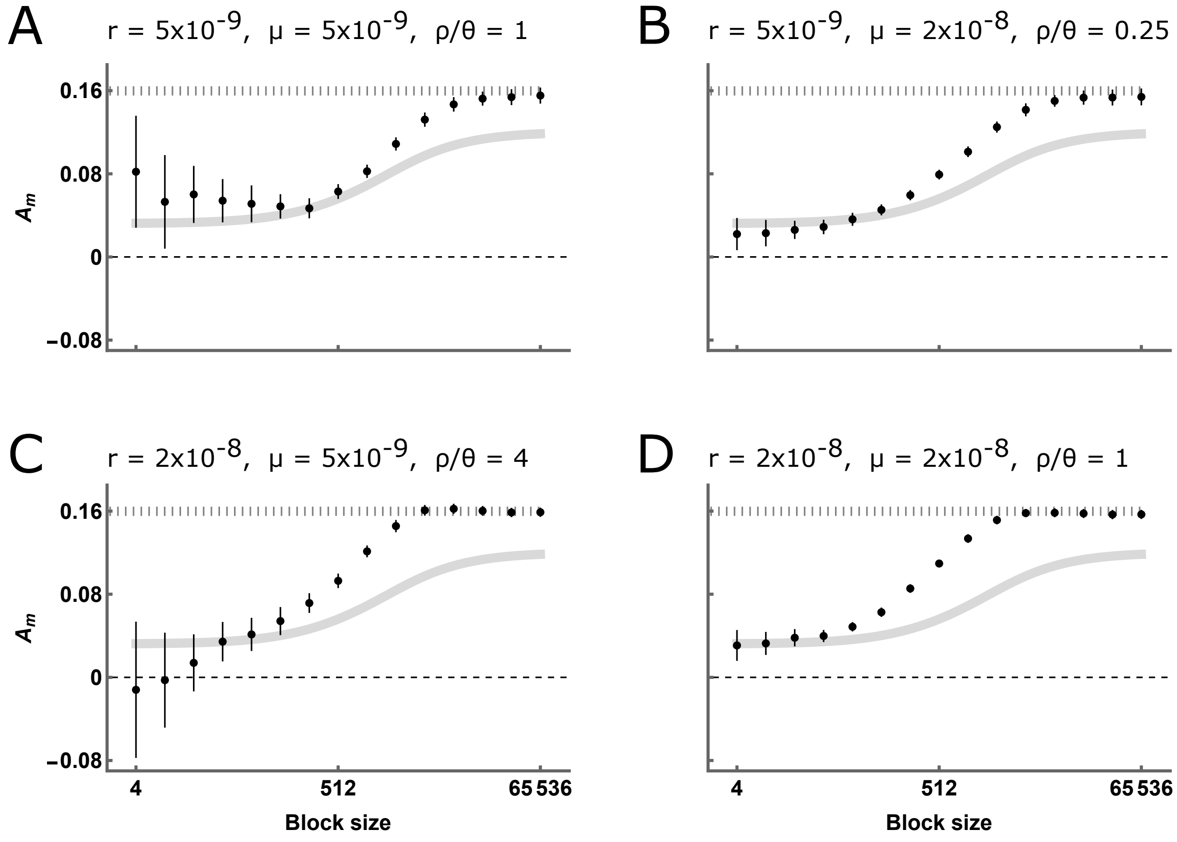

Figure S2: The effect of per-base recombination rate (r) and mutation rate (μ) on estimation of A_m across block sizes. All subplots show estimates of A_m from sequence polymorphisms for a demographic history including gene flow ($N_A = N_B = N_{AB} = 100,000$, $T = 1.0$, $M = 1.0$). All subplots show results for a 197 Mb genome (3000 independently simulated sequences, each with a length of 65 kb). Panels **A** and **B** correspond to low recombination rates, while **C** and **D** show high recombination rates. Panels **A** and **C** correspond to a low mutation rate; panels **B** and **D**, to high. The solid grey line shows the analytic expectation of A_m which assumes no recombination. The dashed gray line shows the expected asymmetry under free recombination. The errors bars correspond to the 95% CIs of each estimate.
